# Rapid genomic changes in *Drosophila melanogaster* adapting to desiccation stress in an experimental evolution system

**DOI:** 10.1186/s12864-016-2556-y

**Published:** 2016-03-15

**Authors:** Lin Kang, Dau Dayal Aggarwal, Eugenia Rashkovetsky, Abraham B. Korol, Pawel Michalak

**Affiliations:** Biocomplexity Institute, Virginia Tech, Blacksburg, VA 24061 USA; Institute of Evolution, University of Haifa, Abba Khoushy Ave 199, Haifa, Israel

**Keywords:** Desiccation stress, Experimental evolution, *Drosophila*, Rapid adaptation, Selective sweep, Evolutionary genomics

## Abstract

**Background:**

Experimental evolution studies, coupled with whole genome resequencing and advances in bioinformatics, have become a powerful tool for exploring how populations respond to selection at the genome-wide level, complementary to genome-wide association studies (GWASs) and linkage mapping experiments as strategies to connect genotype and phenotype. In this experiment, we analyzed genomes of *Drosophila melanogaster* from lines evolving under long-term directional selection for increased desiccation resistance in comparison with control (no-selection) lines.

**Results:**

We demonstrate that adaptive responses to desiccation stress have exerted extensive footprints on the genomes, manifested through a high degree of fixation of alleles in surrounding neighborhoods of eroded heterozygosity. These patterns were highly convergent across replicates, consistent with signatures of ‘soft’ selective sweeps, where multiple alleles present as standing genetic variation become beneficial and sweep through the replicate populations at the same time. Albeit much less frequent, we also observed line-unique sweep regions with zero or near-zero heterozygosity, consistent with classic, or ‘hard’, sweeps, where novel rather than pre-existing adaptive mutations may have been driven to fixation. Genes responsible for cuticle and protein deubiquitination seemed to be central to these selective sweeps. High divergence within coding sequences between selected and control lines was also reflected by significant results of the McDonald-Kreitman and Ka/Ks tests, showing that as many as 347 genes may have been under positive selection.

**Conclusions:**

Desiccation stress, a common challenge to many organisms inhabiting dry environments, proves to be a very potent selecting factor having a big impact on genome diversity.

**Electronic supplementary material:**

The online version of this article (doi:10.1186/s12864-016-2556-y) contains supplementary material, which is available to authorized users.

## Background

While desertification is expected to increase as a result of global climate change and anthropogenic influence, understanding the genomic basis of evolutionary responses to these environmental changes and associated challenges at the organismal level, such as desiccation, is imperative. Organisms are capable of surprisingly rapid adaptations to environmental challenges, such as the application of pesticides [1] or antibiotics [2], suggesting an ample supply of adaptive genetic variation and pervasive impact of positive directional selection. As the environment changes, alleles that previously segregated neutrally, or were only weakly influenced by selection, may become targets of strong selection, in addition to newly arising adaptive mutations. Yet, the precise mode by which beneficial mutations contribute to adaptation remains largely unclear [3].

Recent studies show that adaptations frequently result from ‘soft’ selective sweeps, where multiple adaptive alleles sweep through the population in parallel, either because the alleles were already present as standing genetic variation or arose independently by recurrent de novo mutations [4]. Alternatively, selective sweeps can be ‘hard’, where a novel beneficial allele arises after the onset of selection and sweeps through the population [5]. Whether adaptation originates through hard or soft sweeps depends mostly on the availability of beneficial mutations [6, 7], with hard sweeps taking place when beneficial alleles are absent at the onset of selection and when the waiting time for adaptive mutations to arise is long [4]. Conversely, soft sweeps are more likely to occur when the waiting time until an adaptive mutation arises is shorter than the time it takes for this mutation to spread through the population. In a hard selective sweep, all adaptive alleles coalesce into a single mutation [8], leaving characteristic footprints on neighboring genomic regions, such as a reduction in genetic diversity surrounding the adaptive site [5, 9, 10], a distinct long haplotype [11, 12], and an excess of high-frequency derived alleles and singletons [13-15]. By contrast, soft sweep footprints are less pronounced and therefore more difficult to discern from surrounding neutral polymorphism, since lineages coalesce into two or more alleles, and adaptive loci may remain moderately diverse in the population [16, 17].

A number of approaches have been developed to detect selective sweeps in population genetic data, using a combination of methods based on LD or haplotype structure [17-21], background allele frequency spectrum from Pool-seq data [22], as well as the interplay between recombination and ancestral variation [23]. Haplotype data can also be used to distinguish between signatures of soft and hard sweeps [4, 12, 24]. However, these two might not be easily separable, particularly when hard sweeps are incomplete or when recombination in distant regions linked to hard sweep sites creates patterns of polymorphism that closely mirrors what is expected to be found near soft sweeps, the so-called soft-shoulder effect [25]. These spurious soft sweeps may appear as far as kilobases or even megabases away from the true selected target under moderately strong selection [25].

Experimental evolution uses well-defined selection protocols to force phenotypic divergence, which combined with genome-wide scans (‘evolve-and-resequence’) may narrow down the candidate target regions under positive selection [26–30]. Experimental evolution provides a unique advantage compared to other evolutionary approaches: the ability to replicate an experiment under identical conditions, and thus to distinguish between stochastic and deterministic effects. This replication provides immediate clues about genetic parallelism that can be used to separate soft sweeps from hard sweeps, since sweep signatures parallel between replicates under the same selection pressure, either due to shared standing genetic variation or recurrent mutations, will by definition rule out hard sweeps (although replicate-specific sweep signatures will not automatically preclude soft sweeps). Parallel genetic changes have been observed in experimental evolution experiments with *Escherichia coli* [31–34], yeast [35], *Caenorhabditis elegans* [36], and *Drosophila melanogaster* [26]. While genetic parallelism in the first two systems was due to recurrent mutations, standing genetic variation shared between experimental replicates is the most likely explanation for evolutionary convergence in such organisms as *Drosophila*.

*Drosophila* provide an exceptional model to study the ability to survive droughts, which pose a dramatic ecological and physiological challenge to most organisms inhabiting desert and mesic environments [37]. Xeric-adapted *Drosophila* species are characterized by relatively high desiccation resistance [38, 39], lower water loss rates [37, 38], and decreased metabolic rates under dry conditions [40]. Artificial selection for increased desiccation resistance in *D. melanogaster* leads to critical metabolic alterations, including augmented accumulation of glycogen and differential metabolism of carbohydrates [41]. At the transcriptional level, desiccation stress in *Drosophila mojavensis* alters genes associated with metabolic regulation and carbohydrate metabolism [42], including cuticular hydrocarbons [43].

**Fig. 1:**
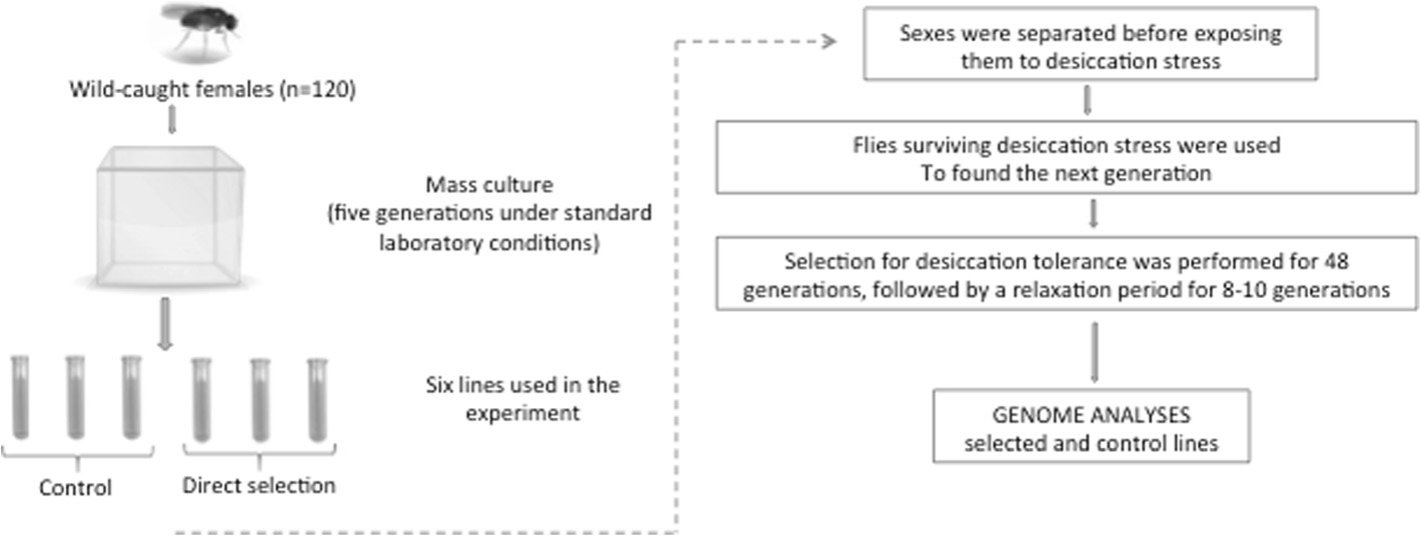
Experimental setup of the experimental evolution experiment for increased desiccation resistance in *Drosophila melanogaster*

In this study we assessed changes in *D. melanogaster* genomes, with the focus on signatures of positive selection, associated with laboratory selection for increased desiccation resistance over 48 generations (Fig. 1). This experimental evolution system resulted in strong adaptive responses leading to increased desiccation resistance and changes in recombination frequencies [44]. Here we show that the selected lines also underwent extensive changes at the genomic level, with a high degree of positive selection-driven sequence divergence and zero or near-zero heterozygosity across numerous sites, producing patterns consistent with selective sweeps.

## Results

### Polymorphism scanning

Three desiccation lines after long-term selection and three parallel control lines (no desiccation) were Illumina-sequenced using pooled genomes per line (Pool-seq). We found a total of 1,217,242 polymorphic sites, 213,819 of which were fixed in all control lines and 603,702 were fixed in all desiccation lines. A total of 993,058, or 81.58 % of the 1,217,242, variants were shared with the Drosophila Genetic Reference Panel lines (DGRP2 [45]). Among the fixed sites in desiccation lines, 83,875 (13.9 %) were in coding regions, compared to 28,539 (13.3 %) fixed sites in coding regions of control lines (Fisher exact test *P* = 2.67 × 10^-10^). A total of 20,185 polymorphic sites were experiment-specific, i.e. one allele fixed in all control lines and an alternative allele fixed in all desiccation lines (Tables 1 and 2, Additional file 1). These SNPs include 745 fixed non-synonymous sites within coding sequences, significantly enriched (relative to all other SNPs in the genome; FDR *P* < 0.05) for functions related to response to copper ion (5 genes), nuclear chromosome part (16 genes), and proteolysis (42 genes) (Additional file 2). We found 19 non-synonymous sites with the same allele fixed in all control lines and two out of the three desiccation lines but with an alternative allele fixed in the remaining desiccation line, a pattern consistent with hard selective sweeps. Examples of these non-synonymous fixed changes occurring in only one desiccation line include chr3L:6319299G > C in olfactory receptor gene *Or65b*, with one of the selection lines, AK2-3, having allele “G” while all other five lines containing “C”, which results in amino acid change from Leu to Phe. Other genes affected by such SNPs include *Ubp64E*, *scny*, *QC*, *Mdr65*, *ndl*, *Cpr72Ec*, and *abd-A* (see Additional file 3 for a complete list). These SNPs tend to be clustered across two islands on chromosome arm 3 L (permutation test *P* = 2.01 × 10^-6^, Additional file 3), suggesting effects of selection.

**Table 1:** Distribution of SNPs

Categories	# of SNPs	% of SNPs	Sequencing coverage	Density of SNP (per Kb)
Intergenic	376,955	30.97	33,640,807	11.21
5′-UTR	32,099	2.64	4,011,958	8.00
CDS (synonymous)	121,477	9.98	22,691,151	7.36
CDS (non-synonymous)	45,577	3.74		
Intron	578,685	47.54	51,989,650	11.13
3′-UTR	50,850	4.18	6,599,093	7.71
ncRNA	11,599	0.95	1,138,125	10.19
Total:	1,217,242	100.00	120,070,784	10.14

**Table 2:** Distribution of fixed SNPs in both control group and desiccation group

Categories	# of SNPs	% of SNPs	Sequencing coverage	Density of SNP (per Kb)
intergenic	6,808	33.73	33,640,807	0.20
5′-UTR	533	2.64	4,011,958	0.13
CDS (synonymous)	2,056	10.19	22,691,151	0.12
CDS (non-synonymous)	745	3.69		
Intron	9,108	45.12	51,989,650	0.18
3′-UTR	755	3.74	6,599,093	0.11
ncRNA	181	0.90	1,138,125	0.16
Total:	20,186	100.00	120,070,784	0.17

### Positive selection signatures

**Fig. 2:**
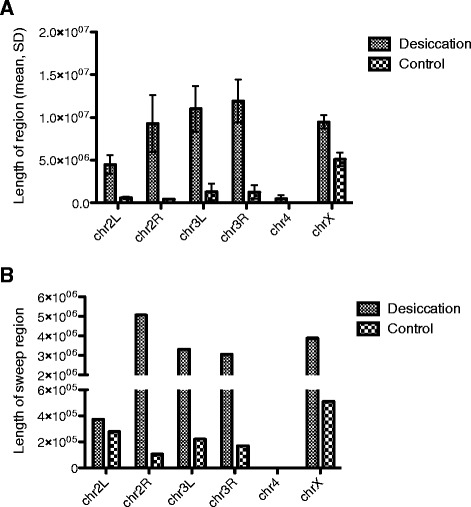
Sweeps found in the Desiccation group and the Control group. Average length of sweep regions (**a**) and length of shared sweep regions (**b**) for Desiccation and Control groups by chromosomes

We used Pool-HMM based on a Hidden Markov Model (HMM) to detect possible selective sweep regions [22]. We found 118, 133, and 121 selective sweep regions in selection/desiccation lines AK2_1, AK2_2, and AK2_3, respectively, and 67 regions were shared among all three of them. By comparison, there were 136, 123, and 103 putative selective sweep regions in control lines AK2_4, AK2_7, and AK2_8, respectively, and 49 regions were shared among all three of them. Even though the sheer number of putative sweep regions was similar between the desiccation group and the control group (chi square test *P* = 0.144), the total length of shared sweep regions among the lines in the desiccation group was 12.2 times longer than the length of shared regions in the control group (15.2 times longer on autosomes and 7.6 times longer on X chromosome, Fig. 2a), and the mean length in the desiccation group was significantly greater than that in the control group (Wilcoxon rank test *P* = 0.031, Fig. 2b). Lower nucleotide diversity is expected within selective sweep regions. To test this prediction, we calculated heterozygosity in 100 kb windows with a step of 10 kb. The overall heterozygosity in the desiccation group was indeed lower compared to heterozygosity of the control group (0.12 ± 0.11 and 0.24 ± 0.08 (mean ± SD) for autosomes in desiccation and control group, respectively; 0.11 ± 0.11 and 0.16 ± 0.11 for X chromosome in desiccation and control group, respectively). The average heterozygosity in shared sweep regions in the desiccation group was 0.02. These results are consistent with a strong selection pressure in the desiccation group, and the lower heterozygosity in X chromosome likely reflects the difference in effective population sizes between X chromosome and autosomes. Simulations of neutral evolution under conditions similar to our experimental setup, based on genomic variation of DGRP2, failed to produce regions of decreased heterozygosity comparable with experimental ones (Additional file 4). Simulated drift effects were also unable to drive the same allele to fixation in three lines while the alternative allele was fixed in the other three lines (there were 745 such non-synonymous sites in the experimental data). When selection was added to the simulations, we were able to generate large genomic regions (~2 Mb) of depleted heterozygosity comparable to observed sweep regions, but only when the selected allele was initially present at a very low frequency (~1/400) and assigned high relative fitness (~4) (Additional file 4).

**Fig. 3:**
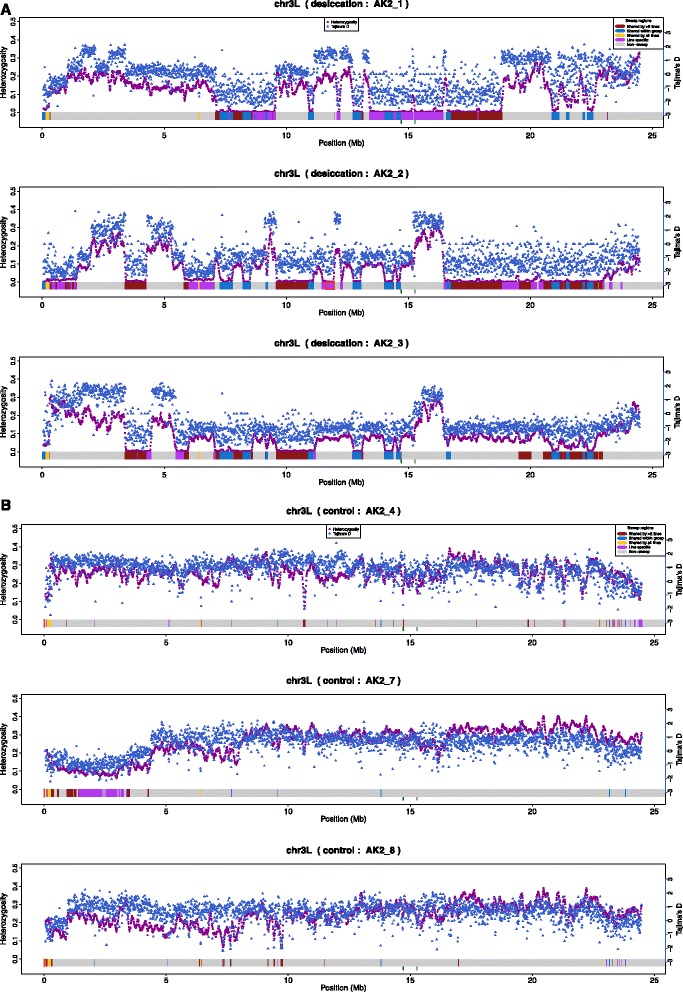
Heterozygosity and Tajima’s D values plotted against the putative selective sweep signatures (horizontal color blocks) along chromosomal arm 3 L in **a** three *D. melanogaster* desiccation lines (AK2_1, AK2_2, AK2_3), and **b** the negative control lines (AK2_4, AK2_7, AK2_8). Horizontal color blocks correspond to putative sweep regions: line-specific (*red*), experimental group –specific (*blue*), shared by all groups (*yellow*), and no sweep (*grey*)

We found blocks of extremely low heterozygosity overlapping with the HMM-identified sweep regions in the desiccation group (Fig. 3). Mean heterozygosity values in non-sweep regions and sweep regions for control lines were 0.238 and 0.144, respectively, and 0.184 and 0.036 for selection lines, respectively. As expected, the reduction in heterozygosity between sweep regions and non-sweep regions was thus stronger in selection lines (Wilcoxon rank sum test *P* = 0.016). Tajima’s D and *π* values produced a very similar pattern to heterozygosity along the sweep regions (Fig. 3, Additional file 5). Accordingly, sweep signatures shared by all lines in the desiccation group likely represent soft sweeps, since the parallelism is most easily explained by preexisting variation at a sufficiently high population frequency to not be stochastically lost by drift. We found a total of 67 putative soft sweep regions (Additional file 6). GO enrichment analysis of genes involved in the shared sweep regions in the desiccation group shows functional enrichments for organophosphate ester transmembrane transporter activity, electron carrier activity, water channel activity, and proteolysis (Additional file 7). On the other hand, line-specific sweep signatures (purple blocks in Fig. 3 and Additional file 5) that are not adjacent to other shared sweep regions and have extremely low heterozygosity values are possible hallmark signatures of hard sweeps based on new mutations. We found five hard sweep candidates (average heterozygosity of 0.007, Fig. 3, Additional files 5 and 8) that contain 435 genes, with enrichment of GO terms related to acetaldehyde dehydrogenase (acetylating) activity, organic cation transmembrane transporter activity, active transmembrane transporter activity, and a variety of metabolic processes (Additional file 9). Similar enrichment patterns were also found in a desiccation resistance study of differentiation expression genes in *Drosophila mojavensis* [42]. A total of 178 genes from the sweep regions shared by our desiccation lines were listed by Rajpurohit et al. [43] as genes differentially expressed in *D. mojavensis* under desiccation stress (Additional File 10).

All of the 19 non-synonymous sites with the same allele fixed in all control lines and two out of the three desiccation lines, but with an alternative allele fixed in the remaining desiccation line, fell within the HMM-identified sweep regions. Seven of the SNPs unique for a single desiccation lines were also located in a sweep region reported only in that line (three genes on chromosome arm 3 L, *Ubp64E*, *scny*, and *Cpr72Ec*, and one gene on 3R, *abd-A*), consistent with a hard sweep pattern. Three of the 19 SNPs from one block (chr2R), containing genes CG30391, CG30393 and CG42672, were located in putative sweep regions shared by all three desiccation lines.

### Positive selection tests with MKT and Ka/Ks

Using the McDonald-Kreitman test (MKT), we found 15 genes likely under positive selection (DoS > 0, *P* < 0.05) and 9 genes under negative selection (DoS < 0, *P* < 0.05) when control and desiccation groups were compared (Table 3). Positively selected genes include *Mucin 68Ca*, an extracellular matrix structural constituent [46], characterized by 11 fixed non-synonymous substitutions between control and desiccation groups. Another gene under positive selection, *foraging* (*for*), is involved in functions related to sucrose response [47], regulation of heart contraction [48], and feeding behavior [49, 50]. *Ninjurin A*, involved in cell adhesion [51] and tissue regeneration [52], is an example of a gene likely under negative selection. Eight of these 15 genes fell in soft sweep regions (Table 3) but none of them in hard sweep regions (see Additional file 10 for the complete list). We also calculated the Ka/Ks ratios using fixed genotypes in each group, and found 347 genes with Ka/Ks ratio greater than 1 (Additional file 10). Among these genes, 277 (79.8 %) showed one fixed non-synonymous substitution- but no fixed synonymous substitution-difference between desiccation and control group. A total of 9 out of 15 genes with positive selection of MKT also had Ka/Ks greater than 1 (Table 3). The 347 genes with Ka/Ks greater than 1 showed a significant enrichment of GO terms related to gastrulation and detection of chemical stimulus (Additional file 11). Although immediate relevance of these GO terms to desiccation is unclear, it is likely that pleiotropic activities of genes involved are more directly related to desiccation. For example, genes belonging to the “gastrulation” GO, such as *sog* (*short gastrulation*), are not only responsible for amnion and serosa development, but also contribute to ectoderm and cuticle formation, with plausible implications for desiccation tolerance [53]. Overall, these results show that selection for desiccation resistance is a very potent evolutionary force leading to adaptive differentiation. To summarize this differentiation, we computed per-site F_ST_ values based on >1 million SNPs. Average pairwise F_ST_ values were 0.081 and 0.110 among selected and control lines, respectively, compared to 0.242 – the average F_ST_ for all selection-control line combinations.

**Table 3:** MKT between control group and desiccation group

#GENEID	DS	DN	PS	PN	DoS	*p*-value (Fisher)
CG2211	2	2	22	0	0.5	0.0185
CG31038^a,b^	0	2	22	4	0.8462	0.0397
CG7213^a^	1	3	14	2	0.625	0.032
CG8545^a,b^	0	4	17	9	0.6538	0.0261
CG8785^a,b^	0	3	18	2	0.9	0.0056
CG9304^a,b^	0	4	27	10	0.7297	0.0099
Cngl	1	2	16	1	0.6078	0.0456
GM130^a^	2	4	25	7	0.4479	0.0466
ir7d^a,b^	1	4	20	6	0.5692	0.0274
Muc68Ca^b^	0	11	29	55	0.3452	0.0166
btsz^a,b^	1	4	75	26	0.5426	0.022
for^b^	1	4	24	0	0.8	0.0002
hay	3	2	21	0	0.4	0.0308
mnd	1	2	14	0	0.6667	0.0221
sog^b^	0	2	20	0	1	0.0043
CG10170	4	1	24	51	-0.48	0.0484
CG13540	2	0	0	8	-1	0.0222
CG15373	4	1	0	4	-0.8	0.0476
CG15394	2	0	0	7	-1	0.0278
CG17734	3	0	0	5	-1	0.0179
CG43798	2	0	2	16	-0.8889	0.0316
Caf1-180	14	3	18	18	-0.3235	0.0355
NijA	11	2	0	3	-0.8462	0.0179
hang	15	0	6	3	-0.3333	0.0415

*DS* synonymous substitution between populations, *DN* non-synonymous substitution between populations, *PS* synonymous polymorphisms within populations, *PN* non-synonymous polymorphisms within populations

^a^falls in putative soft sweep regions

^b^with Ka/Ks ratio greater than 1

## Discussion

We notice that genomic signatures of adaptive responses to directional selection for increased desiccation resistance are highly convergent among selected lines, a pattern consistent with soft selective sweeps operating on standing genetic variation, similar to that found by others [26, 28, 54, 55]. However, unlike Burke et al. [26], who analyzed genomes of *D. melanogaster* selected for accelerated development for over 600 generations, we found numerous sites brought to fixation in the selected lines, including a number of line-specific fixations with signatures of classic, or hard, sweeps in the immediate vicinity. In the selected lines, fixed non-synonymous substitutions shared by all three selected lines were about 40 times more abundant than fixed non-synonymous substitutions limited to only one selected line, and line-convergent signatures of selective sweeps were ~13 times more abundant than line-specific selective sweep signatures. These estimates confirm that albeit soft sweeps are expected to leave more subtle footprints on population genomic data compared with hard sweeps, the former actually emerges as the dominant mode of rapid adaptations in *Drosophila* [4, 26]. These results also suggest that availability of beneficial mutations for rapid adaptations is not a severely limiting factor in *D. melanogaster*. Conversely, hard sweeps are predicted to be dominant only: (i) in consistently small populations; (ii) when adaptation is driven by weak selection in populations of significantly fluctuating size; or (iii) when the adaptive mutation rate is very low, such as when only a specific combination of mutations is adaptive whereas individual mutations are not [4].

We singled out a number of line-specific sweep regions with extremely low heterozygosity or Tajima’s D values, and high fixation rate of the line-unique alleles, including non-synonymous substitutions, as potential hard sweep examples. However, rather than having originated from new beneficial mutations, these putative hard sweep regions, as confirmed by our simulations (Additional file 4), may have actually started from preexisting low-frequency variants unique to a single haplotype background [56]. Under such a scenario the spread and subsequent fixation of the beneficial haplotype could have led to the observed characteristic loss of heterozygosity in a single population, while the beneficial variant might have been lost in the remaining populations due to drift at the low initial frequency, thus resulting in genomic signatures of hard sweeps without the necessity for selection on new mutations. In particular, three genes from chromosome arm 3 L were affected by these sweeps: *Ubp64E*, *scny*, and *Cpr72Ec*. Interestingly, both *Ubp64E* and *scny* are responsible for protein deubiquitination and stabilization [57, 58], whereas both *scny* and *Cpr72Ec* contribute to the structure, growth, and molting of chitin-based cuticle [59, 60]. The ubiquitin-mediated protein degradation has been known to play an important role in desiccation resistance in plants [61] and nematodes [62], while cuticle is instrumental as a barrier against desiccation in terrestrial arthropods, including insects [63]. However, pinpointing true targets of selection, especially in soft sweeps, is very difficult without information about ancestral alleles. We tested Gene Ontology enrichments in the putative sweep regions, but these results only unlikely do represent more than LD effects, or ‘innocent bystanders’ [64], especially in the presence of ‘soft shoulders’ originating from hard sweeps [25].

Similar to our study, but using hypoxic stress for directional selection, Zhou et al. [65] reported significant enrichment for fixed SNPs and fairly large genomic regions to have been affected by adaptive responses to the selection regime. They identified 188 candidate genes, eight of which were previously implicated in hypoxia or similar phenotypes and 12 were linked to the *Notch* pathway. An even more dramatic response to experimental evolution with a high rate of convergent fixation of preexisting alleles was recorded in *C. elegans* [36]. These worm populations were founded with a fixed pair of temperature-sensitive deleterious mutations introgressed into multiple wild genetic backgrounds and allowed to evolve for 50 generations with a mixed mating system. Near-complete fixation, rather than moderate changes in allele frequencies, occurred throughout almost the entire genome in these populations, and entire chromosomal segments became fixed due to strong selection and low effective recombination rates [36].

A selective sweep generates an effectively linked region around the sweeping site, and the region size grows with the strength of positive selection and shrinks with recombination rate [9]. The mode and intensity of selection might also explain why adaptive alleles are driven to fixation in some experimental evolution experiments with *D. melanogaster* ([65] and this study) but not in others [26, 54, 66]. Allele frequencies may plateau without becoming fixed due to heterozygote overdominance or selection on a complex trait with several contributing loci, in which case adaptive allele trajectories are expected to rapidly incline as long as the trait is far away from the fitness optimum, but taper off as the optimum is being approached [67, 68].

Given the fact that we also recorded signatures of selective sweeps in the negative control lines, even though at least 12 times less extensive than in experimental lines, suggests a high rate of false discovery rate, experimental noise or inadvertent laboratory selection. Alternatively, long-range LD in our experimental populations could have arisen as a byproduct of the founders being a small sample of the much larger population, leading to a sampling error and spurious correlations between sites separated by large distances [69]. However, observed at the same time pervasive convergence among experimental replicates suggests that sampling error is likely negligible.

## Conclusions

These results demonstrate the utility of the experimental evolution approach in exploring how patterns of genomic differentiation are shaped by selection for stress resistance. Desiccation, the equilibration of an organism to the relative humidity of the surrounding atmosphere, is an intense stress factor leading to rapid adaptive responses in *Drosophila* under experimental evolution settings. Experimental lines of *D. melanogaster* subject to desiccation stress over 48 generations exhibited rapid resistance increase, accompanied by a high fixation rate and genetically parallel signatures of positive selection in coding sequences, consistent with soft selective sweep patterns.

## Methods

### Flies and the experimental setup

Wild individuals of *D. melanogaster* (*n* = 120) were collected in March 2009 from Madhya Pradesh, Jabalpur, India (23°30’N; 80°01’E; alt. 393 m). Before the start of the selection experiment, mass culture was maintained for 5 generations under standard laboratory conditions at low density (on yeast-cornmeal-agar medium at 21 °C, and ~70 % relative humidity) to eliminate environmental effects. For laboratory selection, virgin flies were sexed under CO_2_ anesthesia at least 48 h prior to the experiment. Then virgin flies (3-4 days old) were placed in groups of 25 into plastic vials containing 2 g of silica gel and covered with discs of foam pieces. Experiments were conducted for males and females separately, since sex has been a factor known to affect desiccation resistance [70, 71]. Flies were subjected to desiccation stress until ~ LT_70_–LT_85_ level of mortality was reached. Control groups were processed in the same manner, excluding desiccation stress. In each generation, we examined ~1000 virgin flies of each sex per replicate, of which at least 100 males and 100 females survived the LT_70-85_ cut-off to become the parents of the next generation. For each group (selection and control), survivors were randomly allocated into three sub-groups (three replicates). The same protocol was recapitulated for 48 generations (each next generation was subjected to analogous treatment), and then selection was relaxed for 8–10 generations before initiating the recombination tests. The control lines were not subjected to any treatment and were maintained in comparable densities to the selection lines on standard media. The influence of starvation stress on mortality rate in the desiccated groups was considered non-significant: in preliminary experiments, flies from the control groups began to die from starvation when all flies from the experimental groups (subjected to combined desiccation + starvation stress) were already dead. Desiccation resistance was measured as the time to lethal dehydration effect under dry air. Groups of 10 female/male flies were placed in dry plastic vials with 2 g of silica gel at the bottom and covered with discs of foam pieces. These vials were then placed in a desiccator chamber. The number of immobile individuals was counted every hour and times to lethal desiccation effects (LT_100_) were recorded. In the present study, we used 3 control and 3 desiccation-resistant lines for sequencing. Average desiccation tolerance of the initial population was 14.8 h and 23.2 h (with SD = 2.88 and 3.44), for males and females, respectively. After 48 generations of selection, these tolerance characteristics increased to 25.3 h and 43.6 h for males and females, respectively, i.e. 3.65SDs and 5.93SDs compared to the starting population.

### Sequencing

In the F_1_ progeny also, we collected 15-20 virgin females and each virgin female was kept into separate small vials together with single brother. The outcome progeny, *n* = 100 females was used for sequencing. Because the F_1_ mothers were reared in separate vials, we achieved our target progeny (*n* = 100 females), mostly from 3-5 F_1_ mother females only. TruSeq 2x100 bp libraries were constructed, barcoded, and sequenced within one lane at 31-38x coverage using Illumina’s HiSeq 2500 (Additional file 12).

### Mapping and genotyping

*D. melanogaster* assembly (dm3) and corresponding annotations (RefSeq) were downloaded from UCSC (http://genome.ucsc.edu/) and used as reference. Raw reads were undergone quality control and filtered through FastqMcf [72]. Clean reads were then mapped to reference using BWA [73] with default parameters. For each line, genotypes were generated using GATK [74] with default parameters except for using ‘--sample_ploidy’ for pooled data and setting heterozygosity (--heterozygosity) to 0.01. Only sites with genotyping quality greater than 30 and minimal depth 10 and maximal depth 250 were kept. Genotypes with more than 2 alleles were discarded from further analyses. For each genotype, percentage of reference allele greater than 99 % or less than 1 % was considered to be homozygous or fixed genotype.

### *F*_*ST*_, π*, θ, Tajima’s D, heterozygosity*

Samtools [75] was used to generate the pileup result. SNPs within 10 bp of indel were discarded and F_ST_ value for each SNP was generated by Poopolation2 [77]. PoPoolation [77] was used to estimate π, Watterson's θ and Tajima’s D with the window size set to 10 Kb. Heterozygosity was calculated based on 100 Kb sliding window with a step of 10 Kb.

### Sweep region identification

Sweeps were identified by Pool-hmm [22], a hidden Markov model for detecting selective sweep based on Pool-Seq data. The parameters used in Pool-hmm were set to be “-n 100 -c 5 -C 400 -q 20 -e sanger -p -k 0.0000000001”, and “--theta” was set to be the *θ* estimated for each sample.

### Neutrality and positive selection simulations

Genome-wide neutrality simulations were performed using forqs [78] to test whether observed patterns of genomic differentiation could result from drift alone. The haplotype data for the simulation under a neutrality model were derived from the Drosophila Genetic Reference Panel 2 (DGRP2, http://dgrp2.gnets.ncsu.edu/) containing 205 inbreed lines [45]. Missing genotypes were set as heterozygous. The mass-breeding phase was simulated for 1,000 generations with a population size of 100,000. Then conditions mimicking our experimental study were simulated with an initial population size of 120, followed by 5 generations of mass culture (population size 2,000), 48 generations of analogous selection (population size 200), 10 generations of analogous relaxed selection, and the final generation with the population size of 100. In total, 6 chromosomes/arms (chr2L, chr2R, chr3L, chr3R, chr4 and chrX) were simulated, and the recombination rate was set to 2. Simulations were repeated several times to mimic the replicas in our study. Heterozygosity was calculated based on the same window size as for the real experimental data. Selection simulations involving a single site on chromosome 2 L were also performed. We used a value of 0.2 as relative fitness (‘FitnessFunction’) for ‘genotype 0’ to represent the ~ LT70–LT85 level of mortality in our experimental desiccation lines. The mass-bred population from the neutrality simulation was used as the initial population (size of 200) and a total of 48 generations were simulated. A range of parameters for fitness and initial allele frequency were tested and each parameter-combination was repeated 10 times (Additional file 4).

### GO Enrichment

To take the gene length into account, an unbiased analysis of gene set enrichment using GOWINDA [79] was performed by considering only SNPs in the CDS regions. Gene mode was used (--mode, assumes all SNPs within a gene are in linkage disequilibrium) with parameters of 1,000,000 simulation (--simulations), the minimum gene number in GO category of 2 (--min-genes) and all SNPs as the background (--snp-file). Enrichment analysis for genes with Ka/Ks greater than 1 was performed on DAVID site (http://david.abcc.ncifcrf.gov/).

### McDonald-Kreitman test and Ka/Ks ratio

Even though McDonald-Kreitman (MK) test was originally designed for testing between species, we use the test in this study to assess adaptive divergence between groups. MK test compares the number of synonymous (Ds) and non-synonymous (Dn) substitutions between species with the number of synonymous (Ps) and non-synonymous (Pn) polymorphisms within species [80]. *P*-values were computed using Fisher exact test. The Ka/Ks ratio, which is the number of non-synonymous substitutions per non-synonymous site (Ka) to the number of synonymous substitutions per synonymous site (Ks), was calculated according to Nei’s approximate method [81].

### Availability of supporting data

The sequence data supporting the results of this article are available in the NCBI’s Sequence Read Archive (SRA) under the accession number SRP066877.

## Additional files

Additional file 1: Table listing the genotype fixed in both control group and desiccation group. (XLSX 2892 kb) Additional file 2: Table listing the enrichment analysis of fixed non-synonymous polymorphic sites. (XLSX 45 kb) Additional file 3: Table listing the putative new mutations in desiccation lines. (XLSX 44 kb) Additional file 4: Figures illustrating neutrality-selection simulation results. (A-F) Heterozygosity values along chromosomes from simulated neutral evolution. (G-H) Average sizes of regions with low heterozygosity values for different parameter combinations under a selection simulation. (I) Examples of heterozygosity values along chromosome 2 L from a selection simulation. (PDF 541 kb) Additional file 5: Figures of heterozygosity and Tajima’s D values plotted against the putative selective sweep signatures (horizontal color blocks) along chromosomal 2 L, 2R, 3R, and X. (PDF 2138 kb) Additional file 6: Table listing the regions of putative soft selection sweeps. (XLSX 41 kb) Additional file 7: Table listing the enrichment analysis of genes in putative soft sweeps. (XLSX 72 kb) Additional file 8: Table listing the regions of putative hard selection sweeps. (XLSX 32 kb) Additional file 9: Table listing the enrichment analysis of genes in putative hard sweeps. (XLSX 41 kb) Additional file 10: Table listing the MKT and Ka/Ks for all genes. (XLSX 997 kb) Additional file 11: Table listing the enrichment analysis of genes with Ka/Ks ratio greater than 1. (XLSX 36 kb) Additional file 12: Table listing the statistics of clean sequencing reads. (XLSX 35 kb)

## Abbreviations

DGRP2: Drosophila Genetic Reference Panel 2
Dn: the number of non-synonymous substitutions between species
DoS: direction of selection
Ds: the number of synonymous substitutions between species
FDR: false discovery rate
Ka: the number of non-synonymous substitutions per non-synonymous site
Ks: the number of synonymous substitutions per synonymous site
MKT: McDonald-Kreitman test
Pn: the number of non-synonymous polymorphisms
Ps: the number of synonymous polymorphisms
