# Supplementary material for: Rapid genomic changes in Drosophila melanogaster adapting to desiccation stress in an experimental evolution system
Source: BMC Genomics. 2016 Mar 15;17:233. doi: 10.1186/s12864-016-2556-y (PMC4791783; doi:10.1186/s12864-016-2556-y)
Supplement: Additional file 4: — Figures illustrating neutrality-selection simulation results. (A-F) Heterozygosity values along chromosomes from simulated neutral evolution. (G-H) Average sizes of regions with low heterozygosity values for different parameter combinations under a selection simulation. (I) Examples of heterozygosity values along chromosome 2 L from a selection simulation. (PDF 541 kb) [file 12864_2016_2556_MOESM4_ESM.pdf]

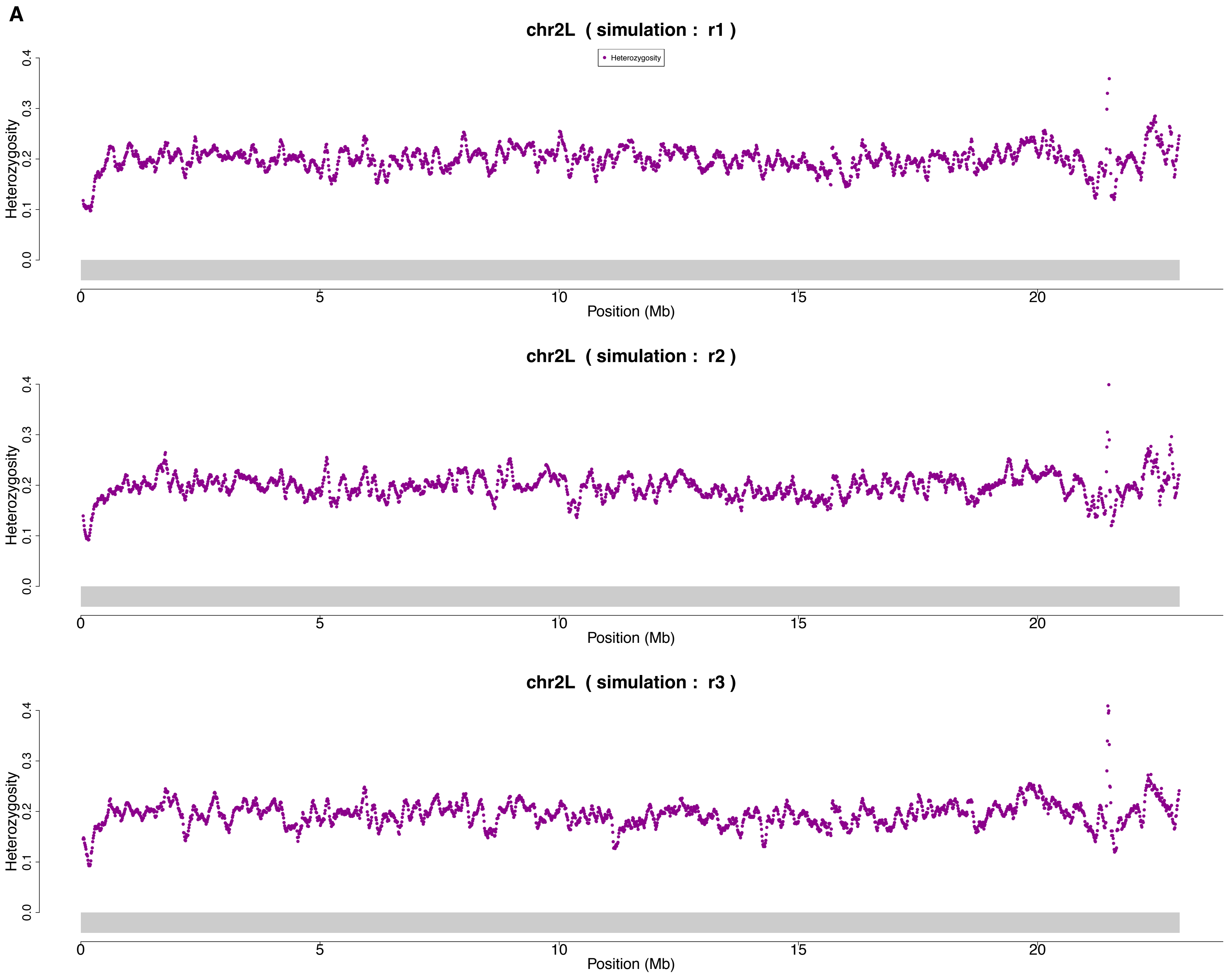

A
chr2L (simulation : r1)
Heterozygosity
Heterozygosity
0.0
0.1
0.2
0.3
0.4
0
5
10
15
20
Position (Mb)
chr2L (simulation : r2)
Heterozygosity
0.0
0.1
0.2
0.3
0.4
0
5
10
15
20
Position (Mb)
chr2L (simulation : r3)
Heterozygosity
0.0
0.1
0.2
0.3
0.4
0
5
10
15
20
Position (Mb)

B

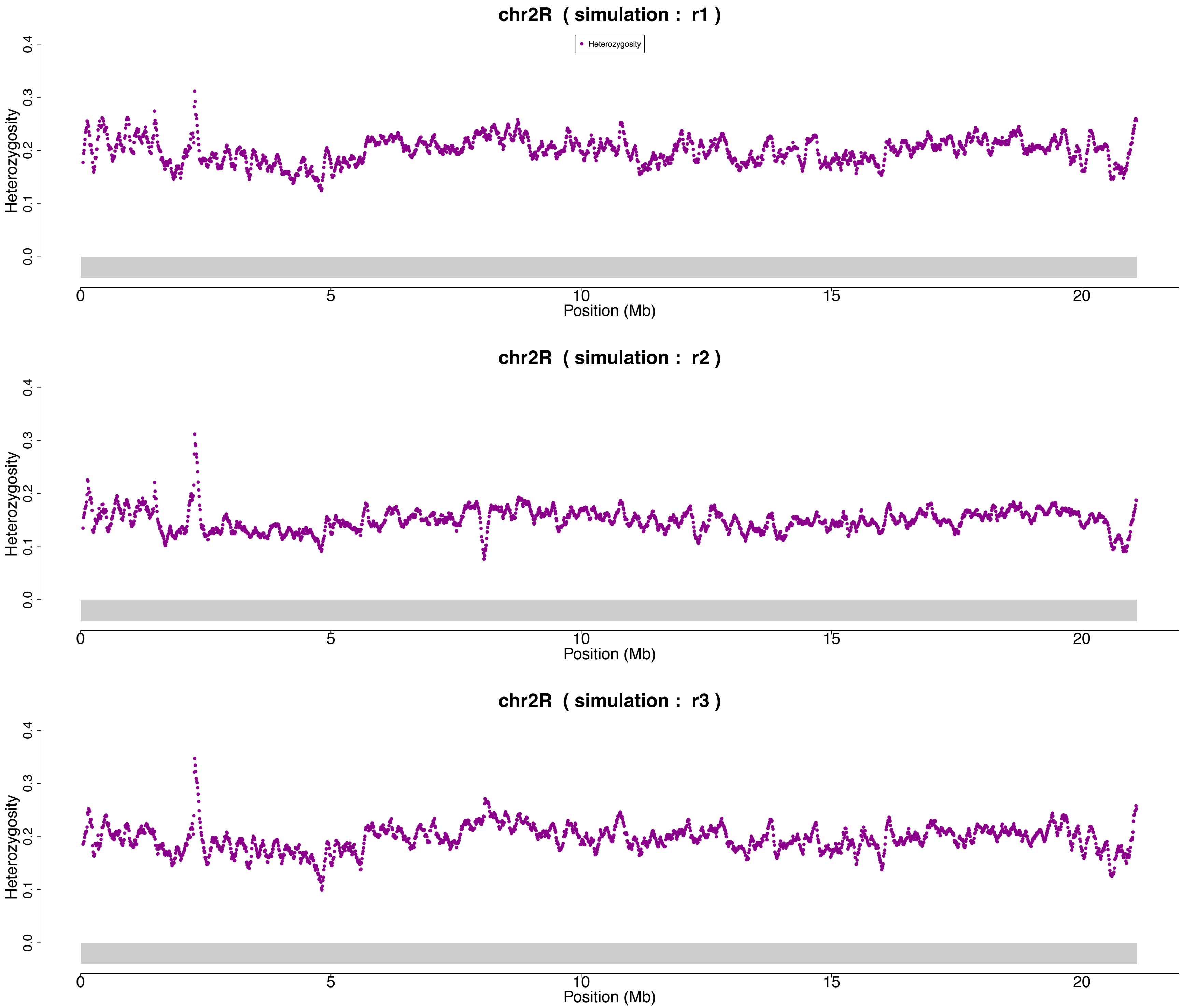
chr2R (simulation : r1)
Heterozygosity
Heterozygosity
0.0
0.1
0.2
0.3
0.4
0
5
10
15
20
Position (Mb)
chr2R (simulation : r2)
Heterozygosity
0.0
0.1
0.2
0.3
0.4
0
5
10
15
20
Position (Mb)
chr2R (simulation : r3)
Heterozygosity
0.0
0.1
0.2
0.3
0.4
0
5
10
15
20
Position (Mb)

C

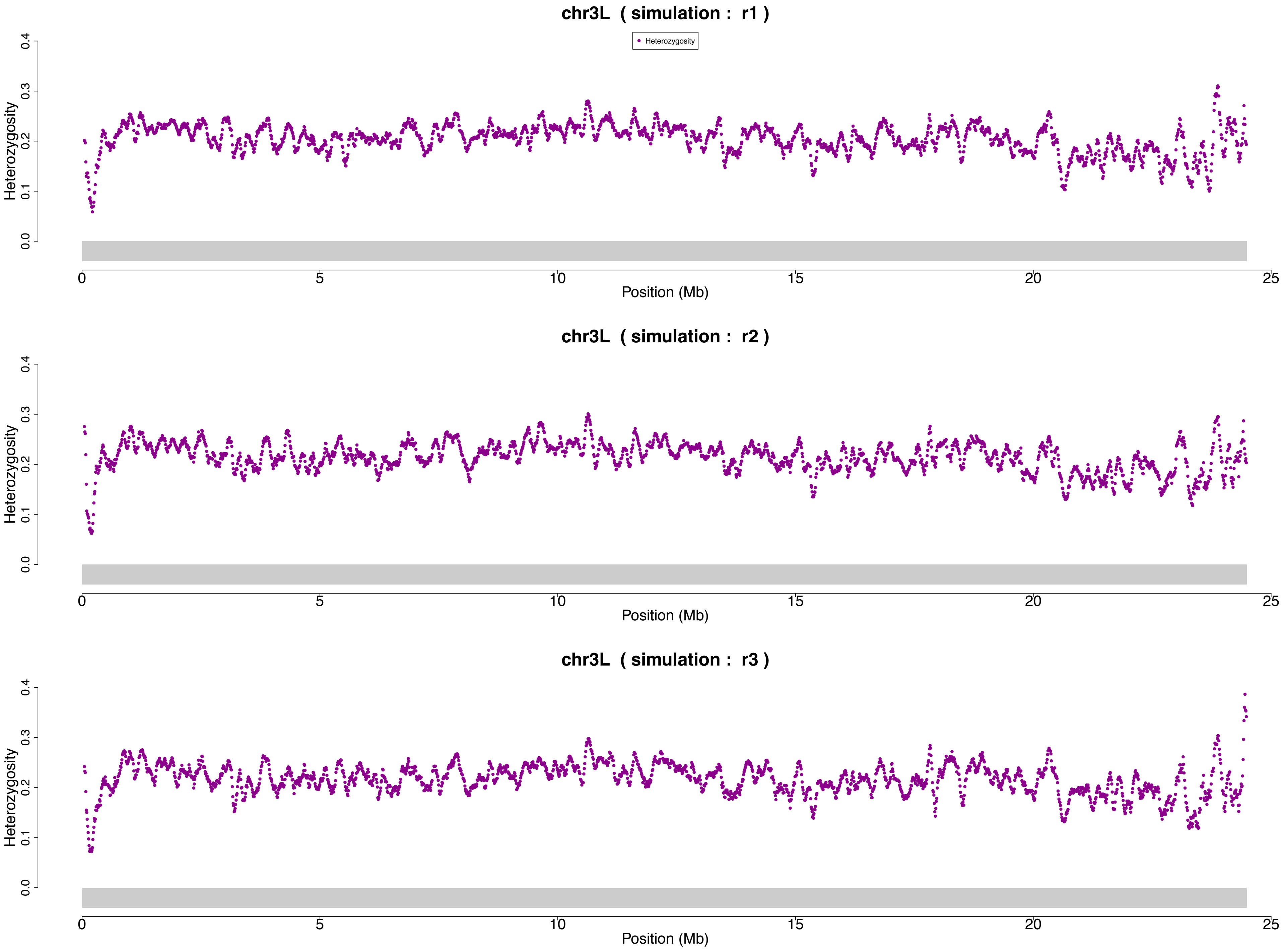

chr3L (simulation : r1)
Heterozygosity
Heterozygosity
0.0
0.1
0.2
0.3
0.4
0
5
10
15
20
25
Position (Mb)
chr3L (simulation : r2)
Heterozygosity
0.0
0.1
0.2
0.3
0.4
0
5
10
15
20
25
Position (Mb)
chr3L (simulation : r3)
Heterozygosity
0.0
0.1
0.2
0.3
0.4
0
5
10
15
20
25
Position (Mb)

D

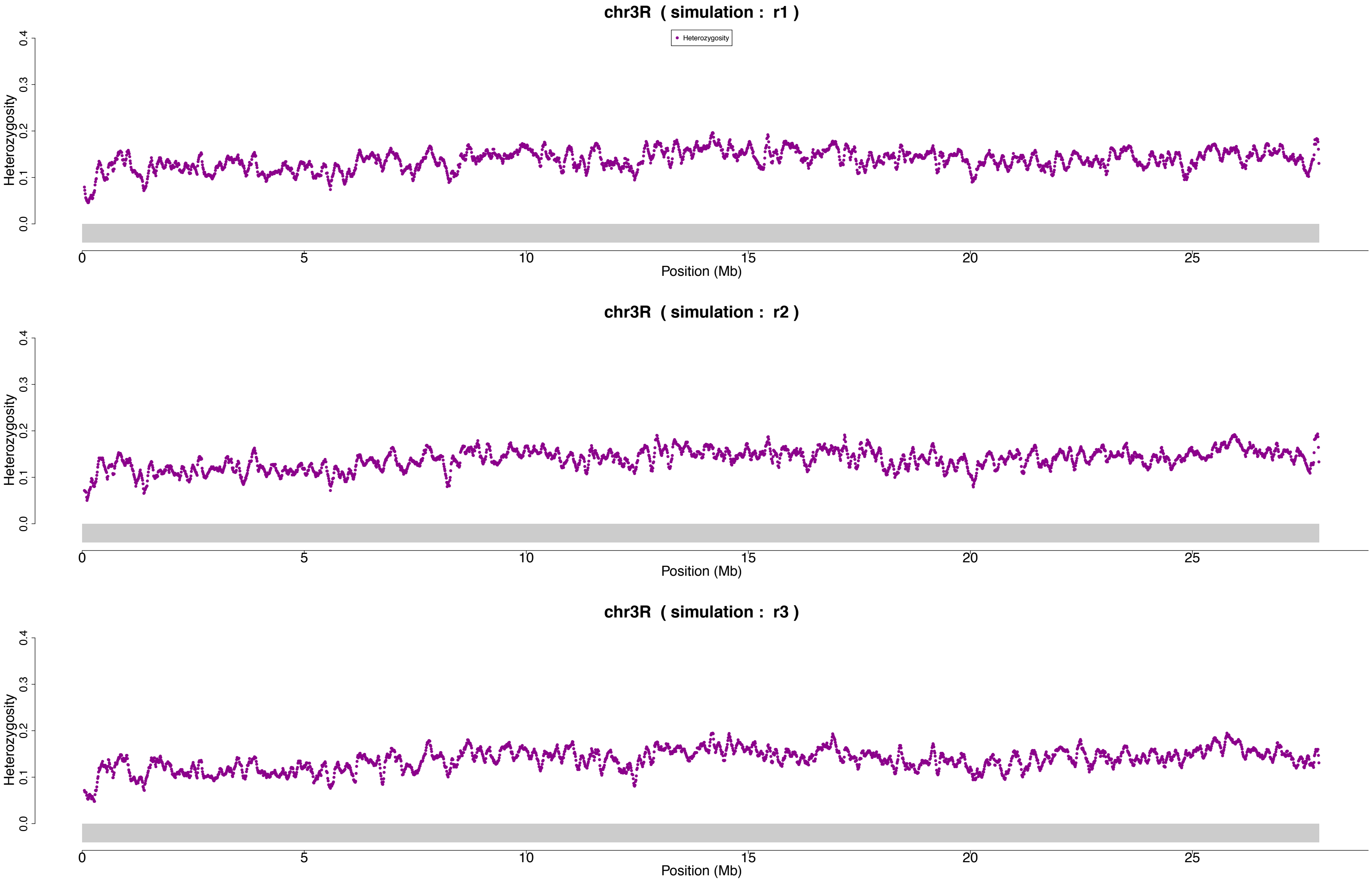
chr3R (simulation : r1)
Heterozygosity
Heterozygosity
0.0
0.1
0.2
0.3
0.4
0
5
10
15
20
25
Position (Mb)
chr3R (simulation : r2)
Heterozygosity
0.0
0.1
0.2
0.3
0.4
0
5
10
15
20
25
Position (Mb)
chr3R (simulation : r3)
Heterozygosity
0.0
0.1
0.2
0.3
0.4
0
5
10
15
20
25
Position (Mb)

E

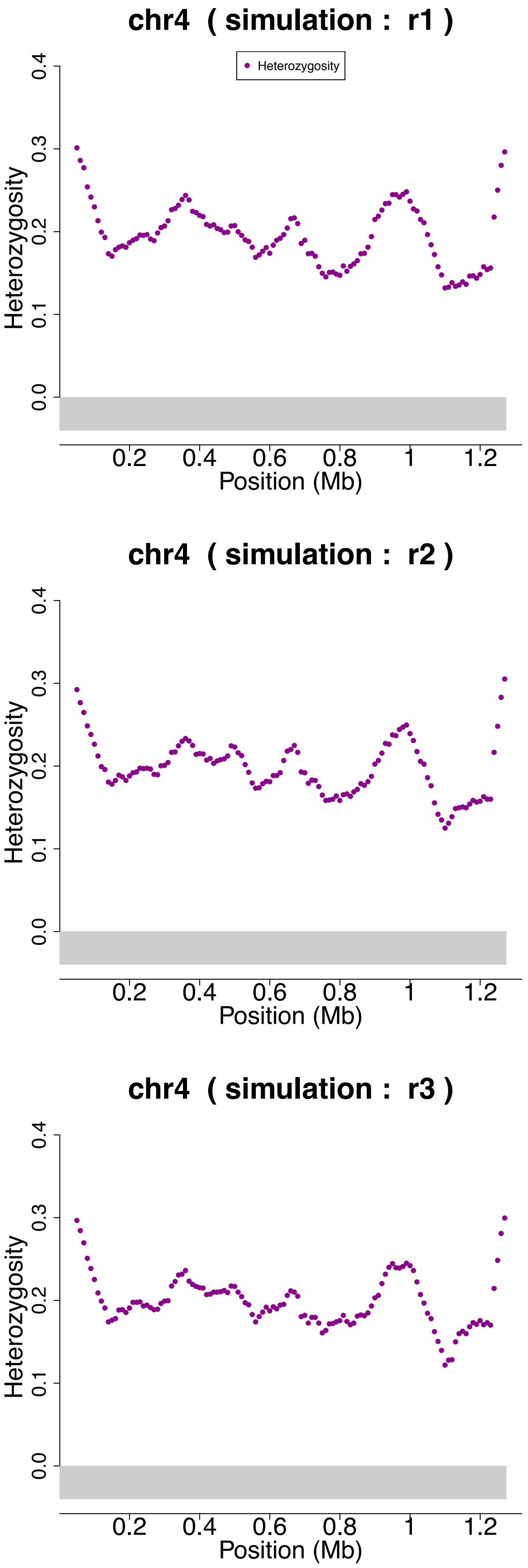

chr4 (simulation : r1)
Heterozygosity
0.0
0.1
0.2
0.3
0.4
0.2
0.4
0.6
0.8
1
1.2
Position (Mb)
chr4 (simulation : r2)
Heterozygosity
Position (Mb)
chr4 (simulation : r3)
Heterozygosity
Position (Mb)

F

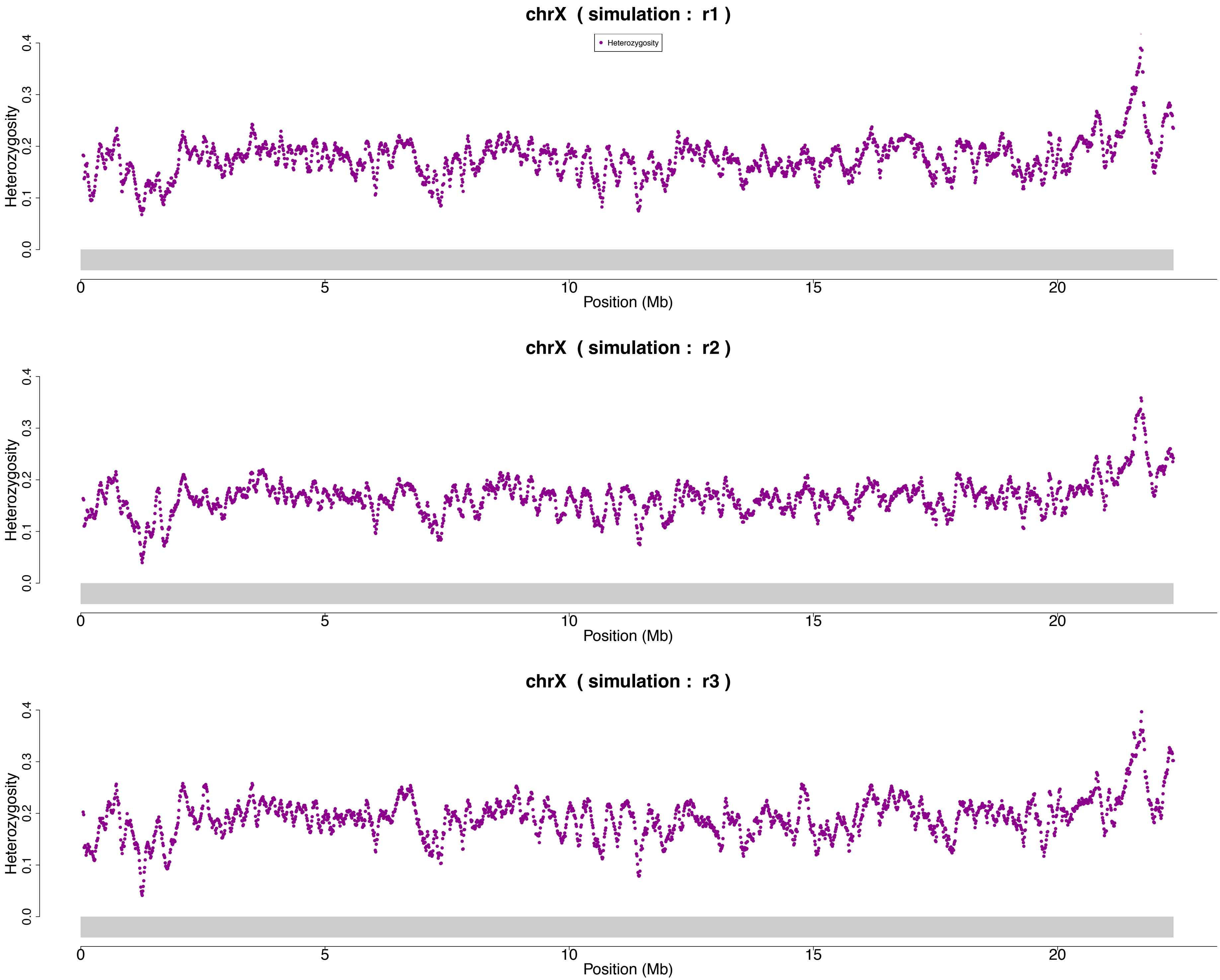
chrX (simulation : r1)
Heterozygosity
Heterozygosity
0.0
0.1
0.2
0.3
0.4
0
5
10
15
20
Position (Mb)
chrX (simulation : r2)
Heterozygosity
0.0
0.1
0.2
0.3
0.4
0
5
10
15
20
Position (Mb)
chrX (simulation : r3)
Heterozygosity
0.0
0.1
0.2
0.3
0.4
0
5
10
15
20
Position (Mb)

G

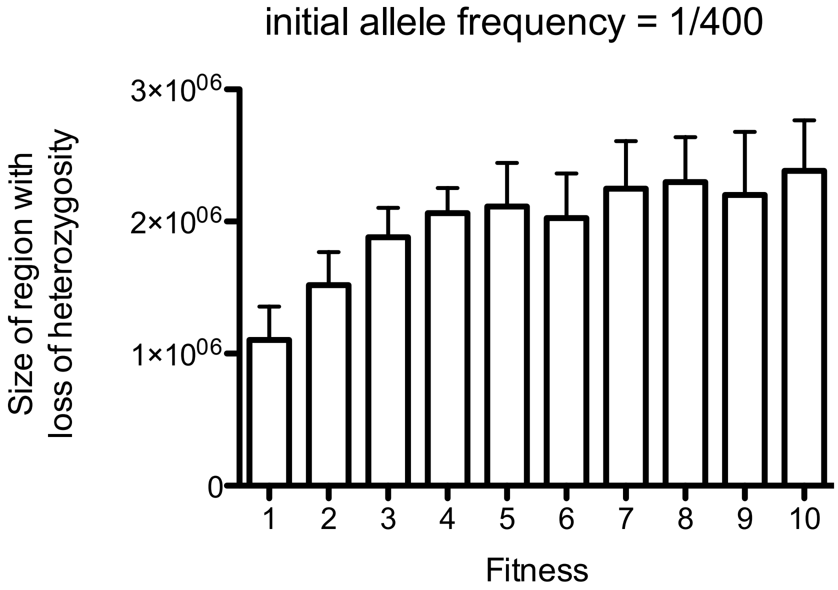

initial allele frequency = 1/400
Size of region with loss of heterozygosity
3×10^06
2×10^06
1×10^06
0
1 2 3 4 5 6 7 8 9 10
Fitness

H

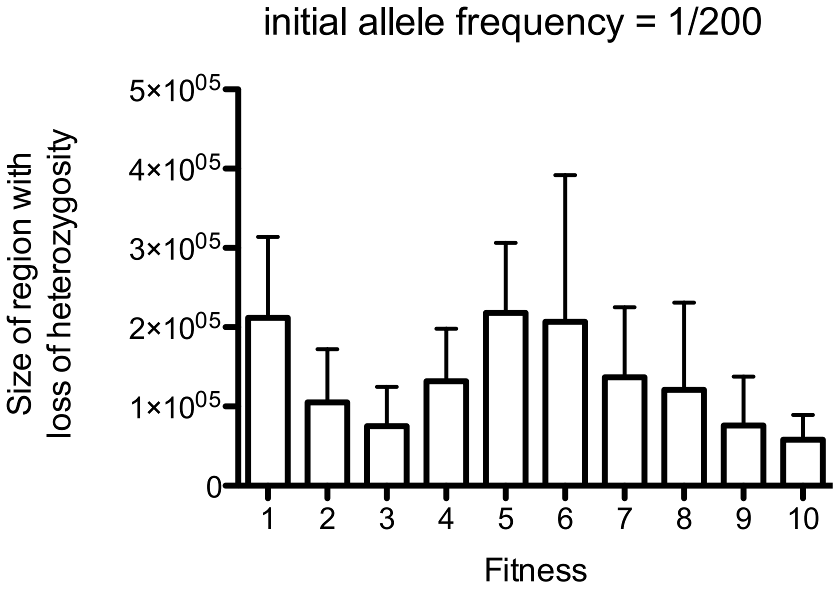

initial allele frequency = 1/200
Size of region with loss of heterozygosity
5×10^05
4×10^05
3×10^05
2×10^05
1×10^05
0
1 2 3 4 5 6 7 8 9 10
Fitness

I

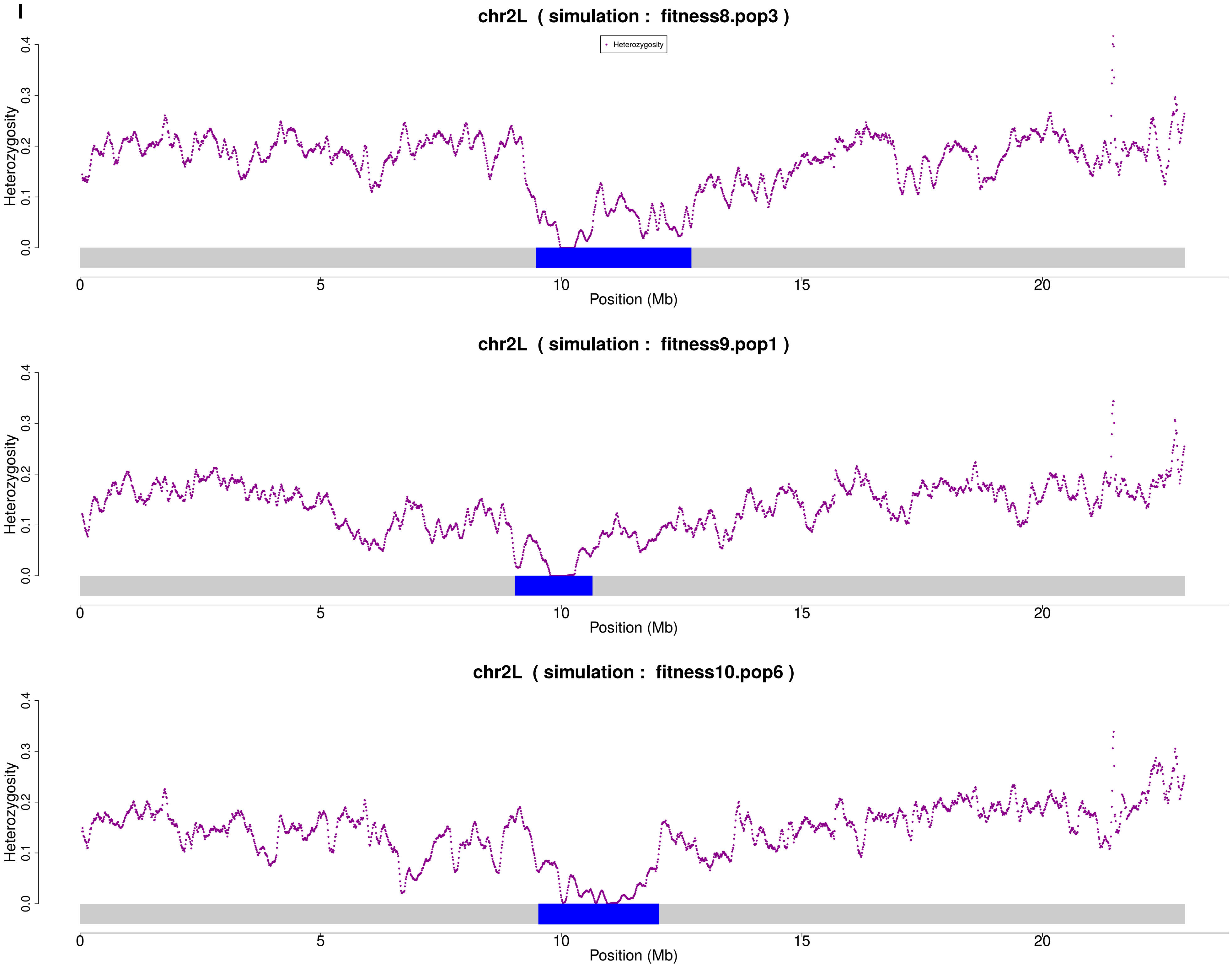
chr2L (simulation : fitness8.pop3)
Heterozygosity
Heterozygosity
0.0
0.1
0.2
0.3
0.4
0
5
10
15
20
Position (Mb)
chr2L (simulation : fitness9.pop1)
Heterozygosity
0.0
0.1
0.2
0.3
0.4
0
5
10
15
20
Position (Mb)
chr2L (simulation : fitness10.pop6)
Heterozygosity
0.0
0.1
0.2
0.3
0.4
0
5
10
15
20
Position (Mb)
