# Supplementary material for: Rapid genomic changes in Drosophila melanogaster adapting to desiccation stress in an experimental evolution system
Source: BMC Genomics. 2016 Mar 15;17:233. doi: 10.1186/s12864-016-2556-y (PMC4791783; doi:10.1186/s12864-016-2556-y)

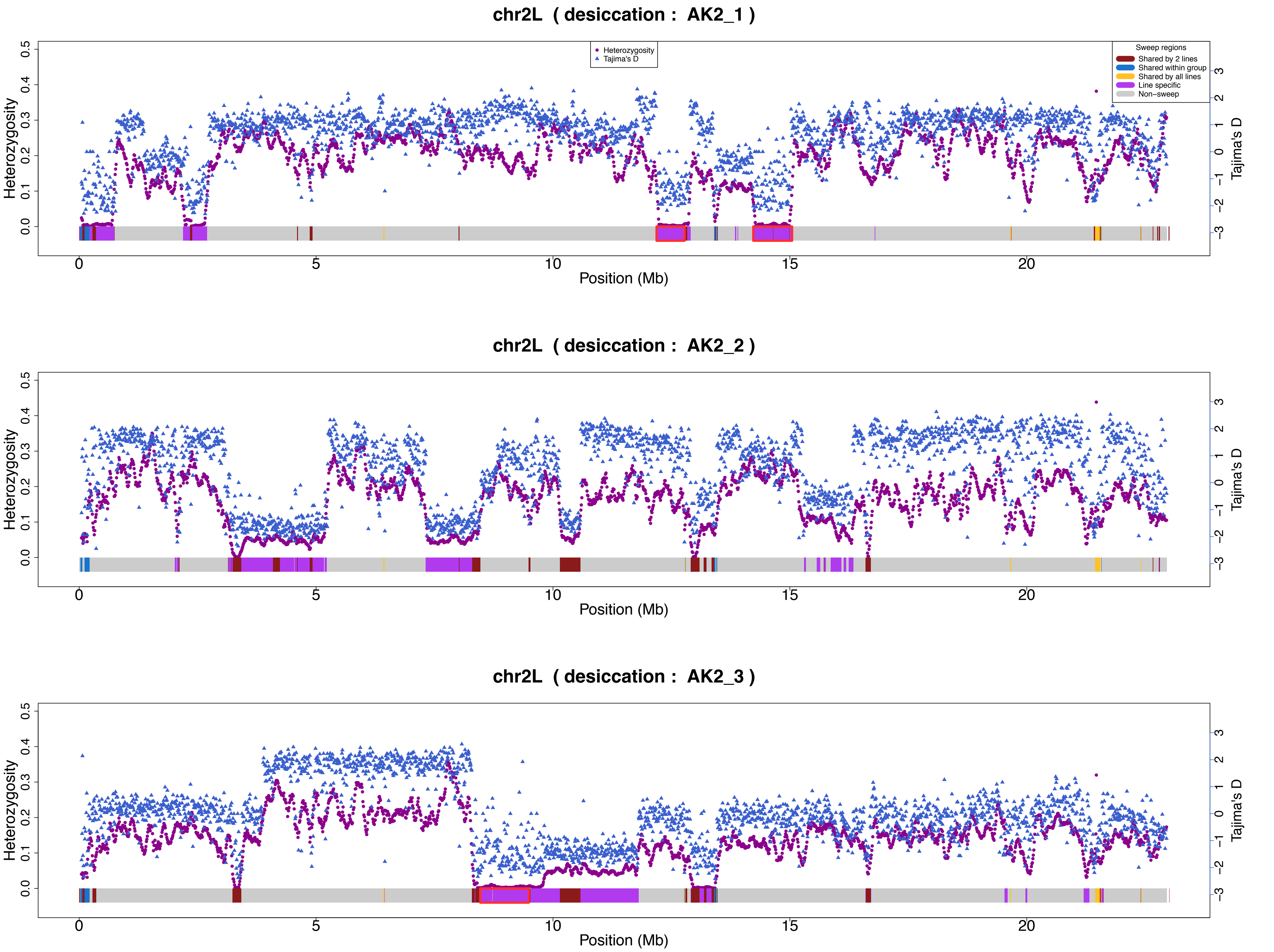

chr2L (desiccation : AK2_1)
Heterozygosity
Tajima's D
Sweep regions
Shared by 2 lines
Shared within group
Shared by all lines
Line specific
Non−sweep
Heterozygosity
Position (Mb)
chr2L (desiccation : AK2_2)
Heterozygosity
Tajima's D
Position (Mb)
chr2L (desiccation : AK2_3)
Heterozygosity
Tajima's D
Position (Mb)

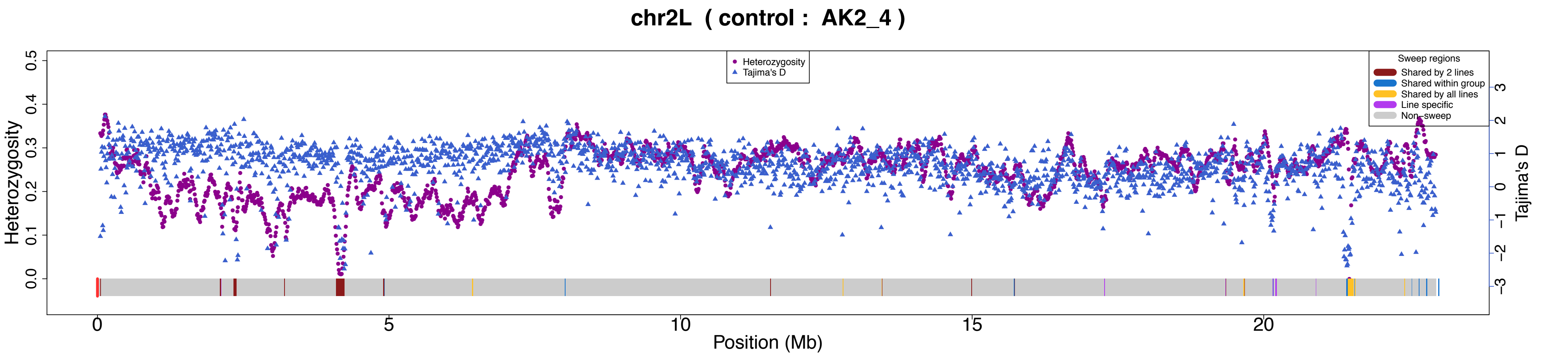
chr2L (control : AK2_4)
Heterozygosity
Tajima's D
Sweep regions
Shared by 2 lines
Shared within group
Shared by all lines
Line specific
Non-sweep
Position (Mb)

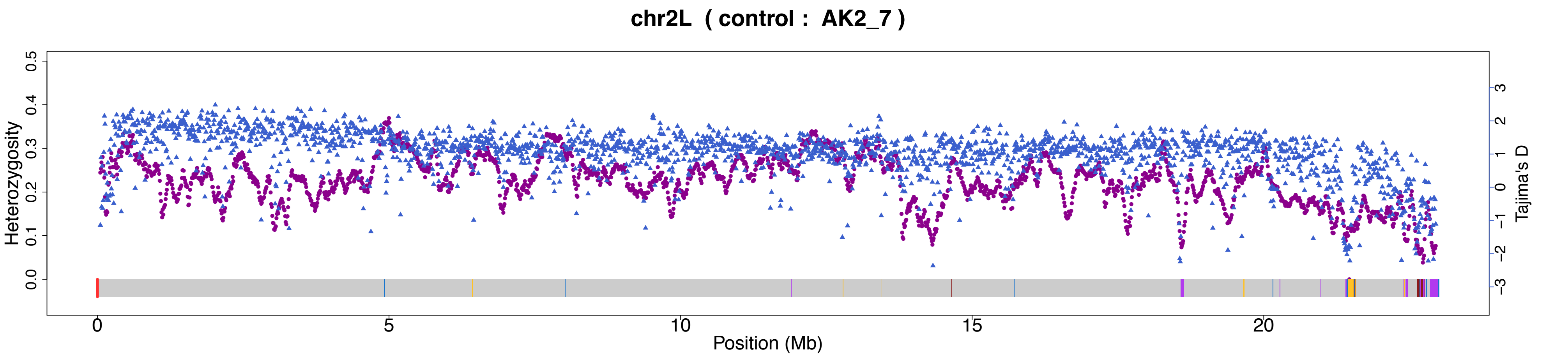
chr2L (control : AK2_7)
Heterozygosity
Tajima's D
Position (Mb)

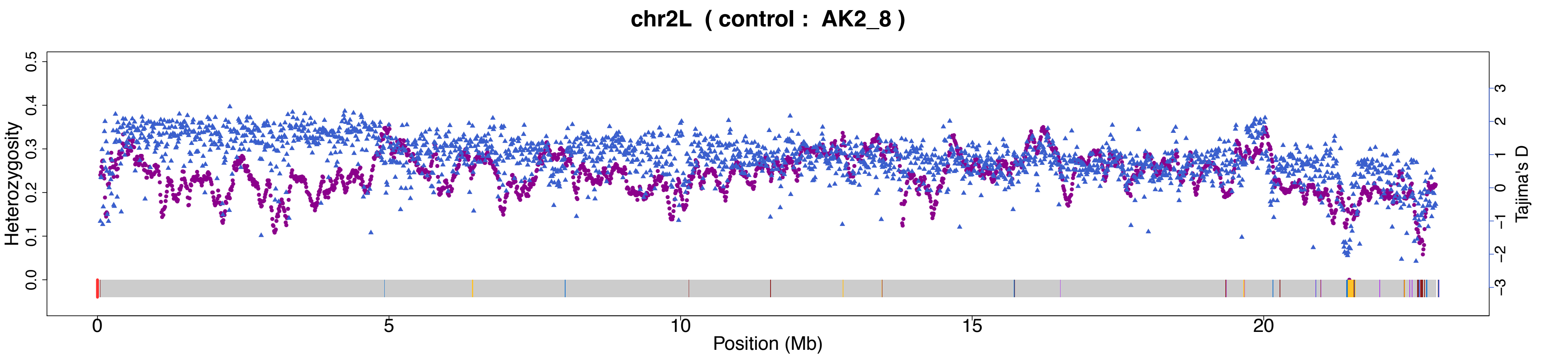
chr2L (control : AK2_8)
Heterozygosity
Tajima's D
Position (Mb)

chr2R (desiccation : AK2_1)

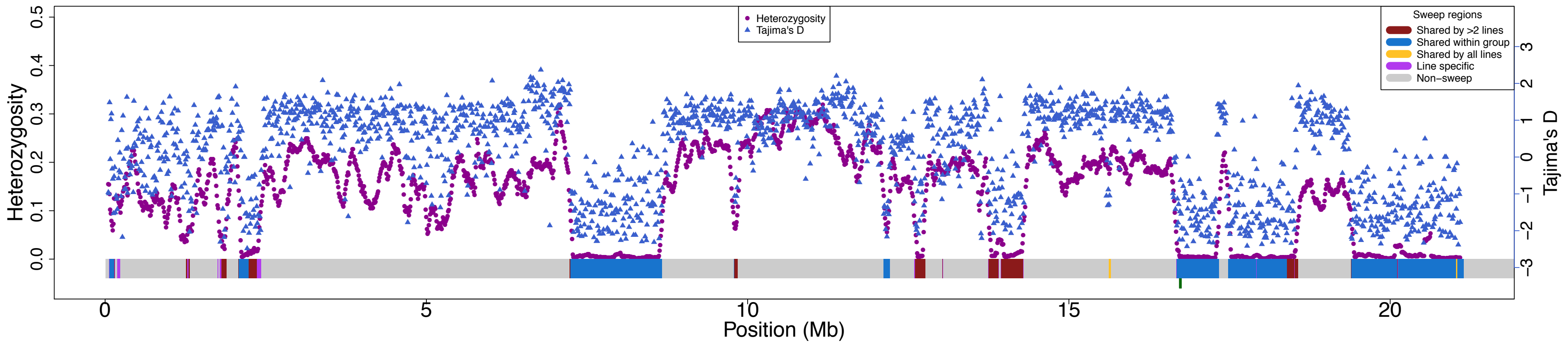

chr2R (desiccation : AK2_2)

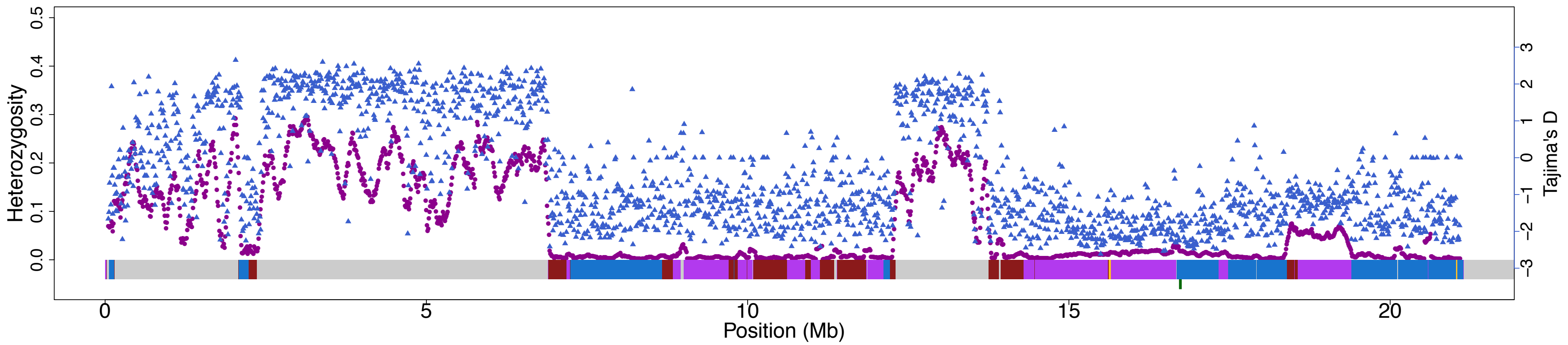

chr2R (desiccation : AK2_3)

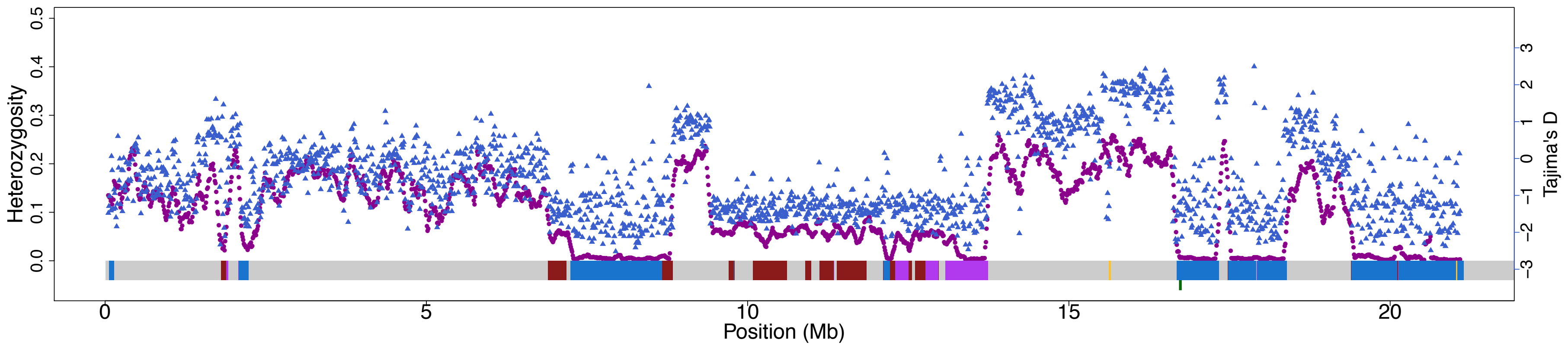

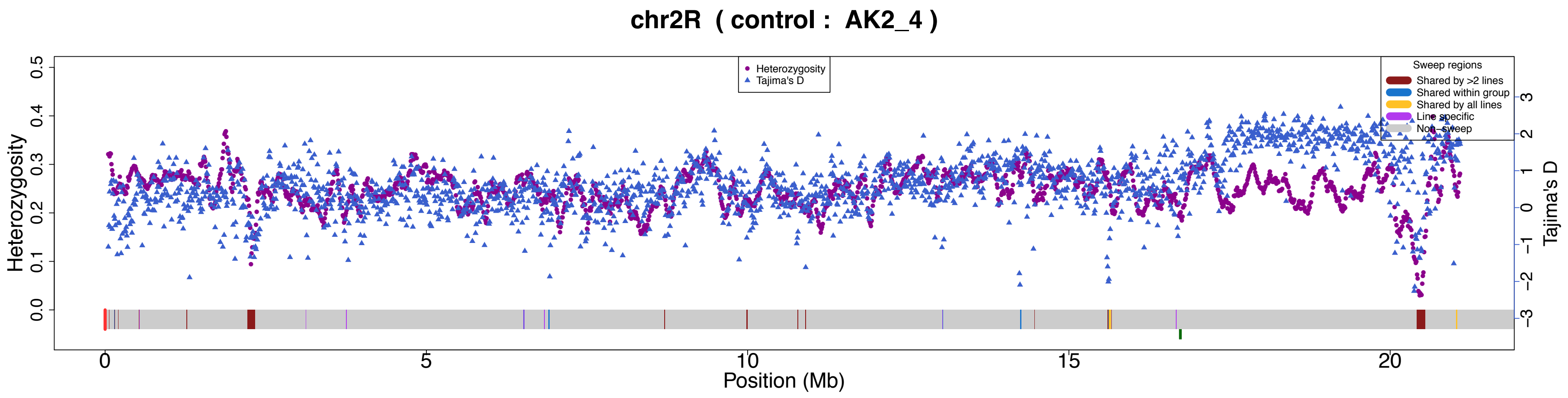
chr2R (control : AK2_4)
Heterozygosity
Tajima's D
Sweep regions
Shared by >2 lines
Shared within group
Shared by all lines
Line specific
Non-sweep
Position (Mb)

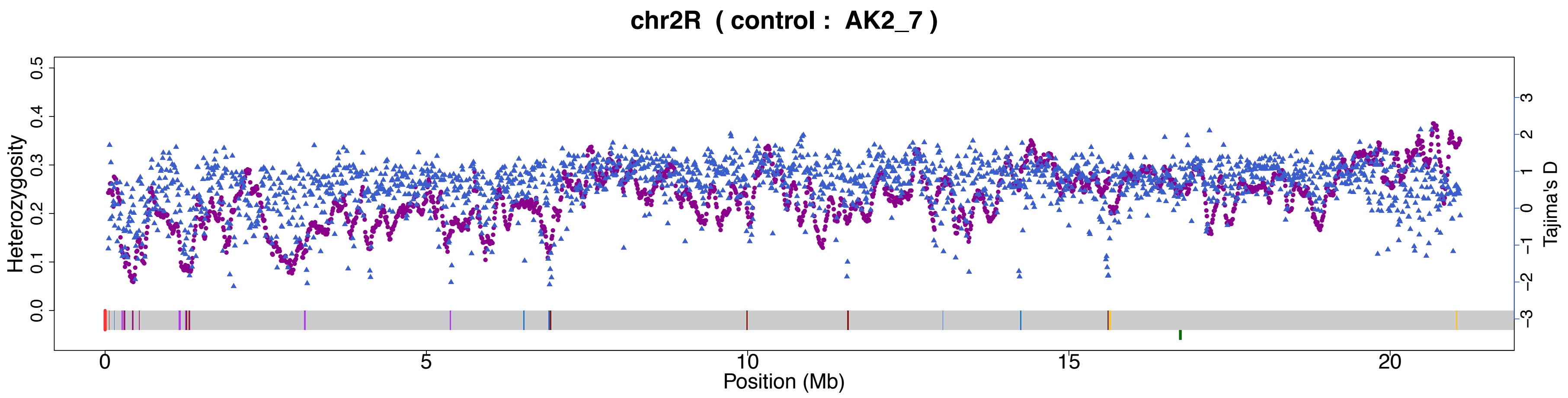
chr2R (control : AK2_7)
Heterozygosity
Tajima's D
Position (Mb)

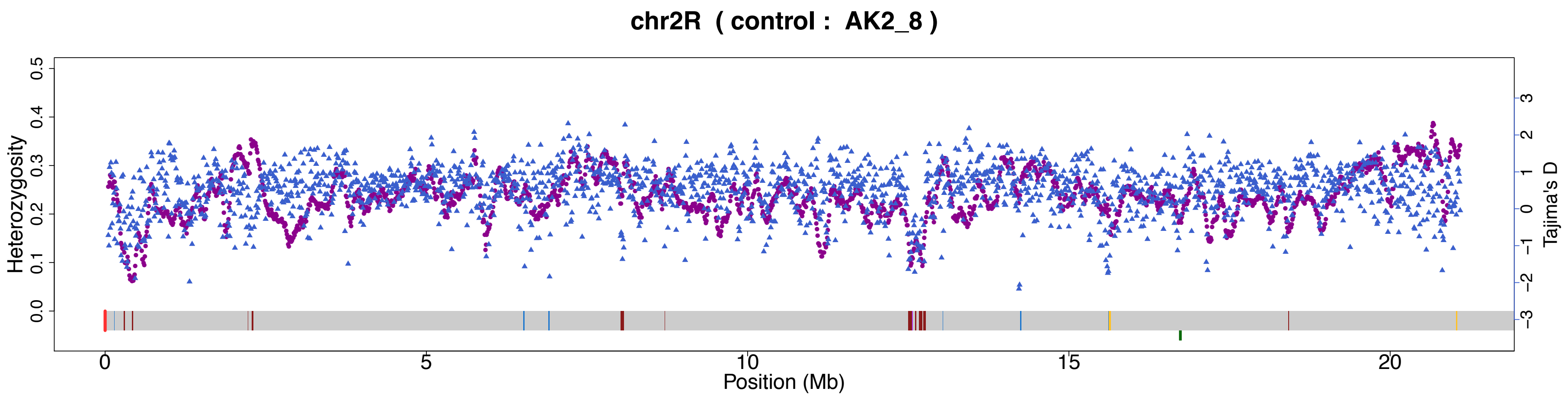
chr2R (control : AK2_8)
Heterozygosity
Tajima's D
Position (Mb)

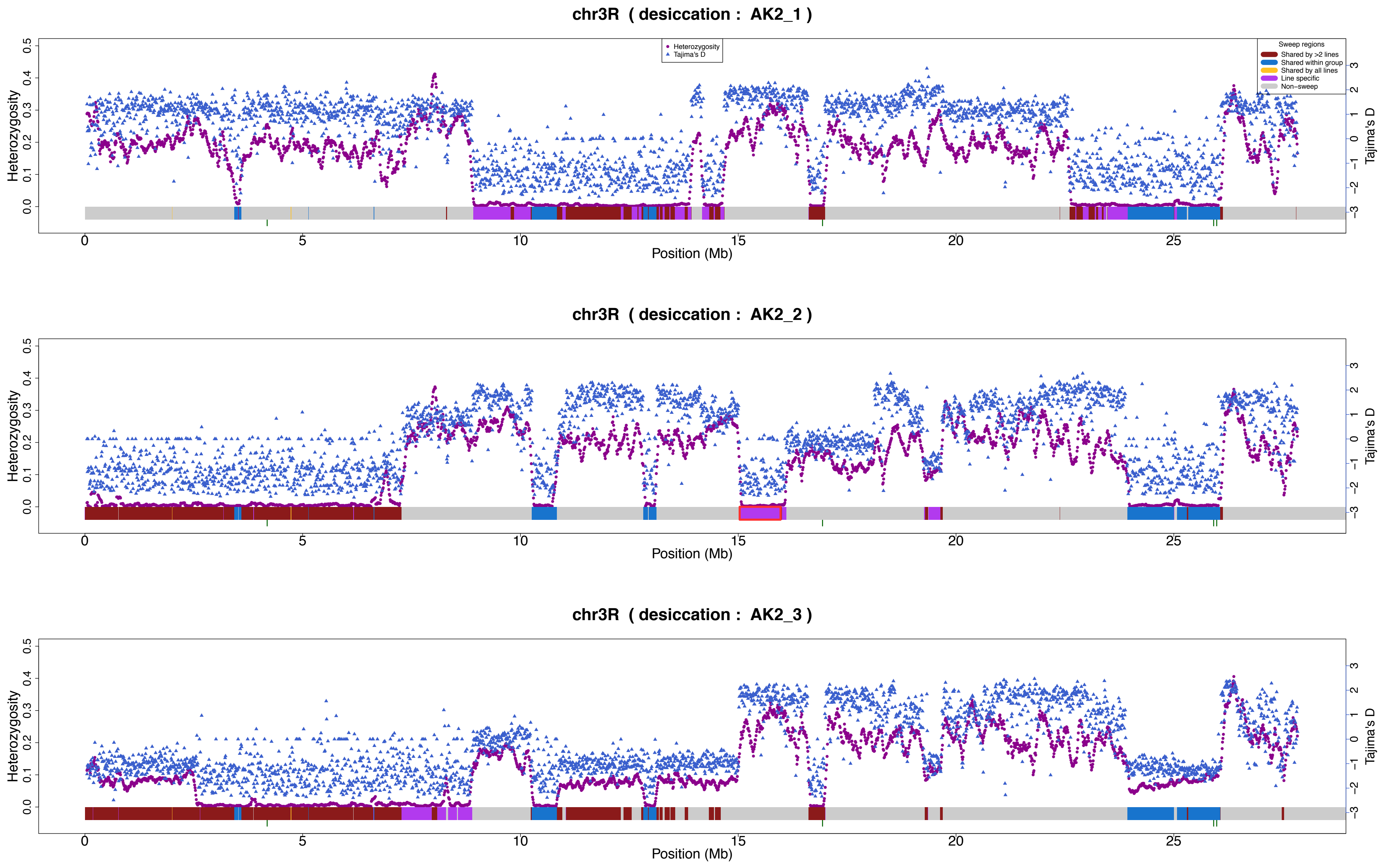
chr3R (desiccation : AK2_1)
Heterozygosity
Tajima's D
Sweep regions
Shared by >2 lines
Shared within group
Shared by all lines
Line specific
Non−sweep
Position (Mb)
chr3R (desiccation : AK2_2)
Heterozygosity
Tajima's D
Position (Mb)
chr3R (desiccation : AK2_3)
Heterozygosity
Tajima's D
Position (Mb)

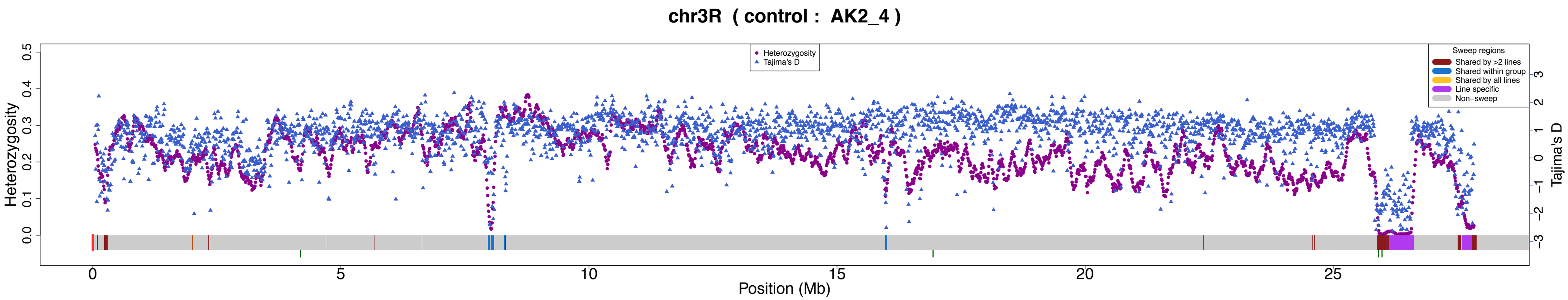
chr3R (control : AK2_4)
Heterozygosity
Tajima's D
Sweep regions
Shared by >2 lines
Shared within group
Shared by all lines
Line specific
Non−sweep
Position (Mb)

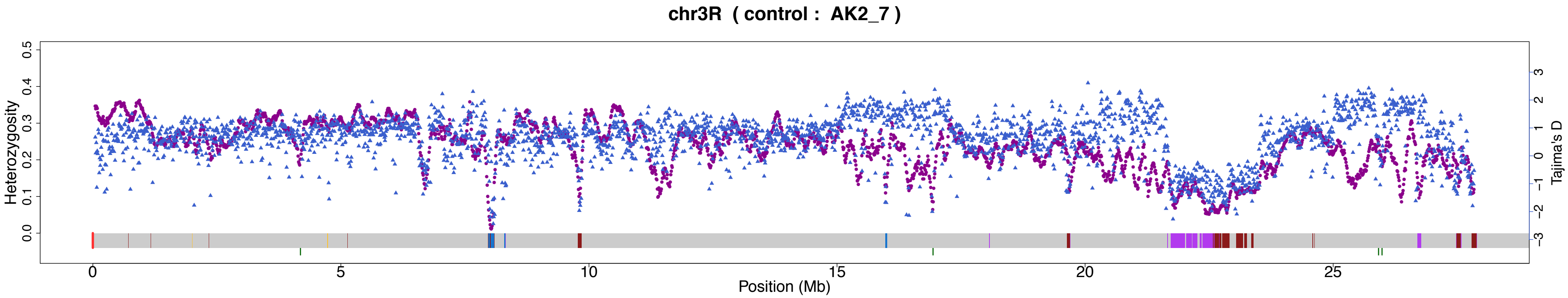
chr3R (control : AK2_7)
Heterozygosity
Tajima's D
Position (Mb)

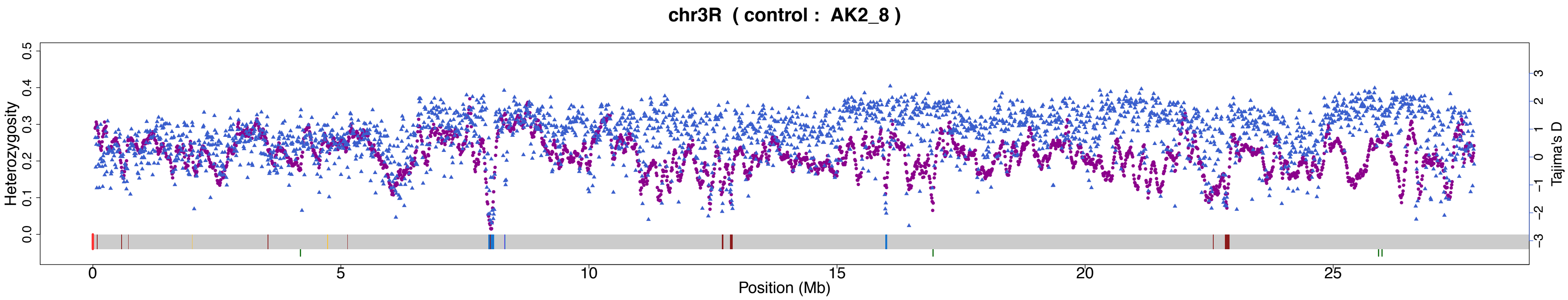
chr3R (control : AK2_8)
Heterozygosity
Tajima's D
Position (Mb)

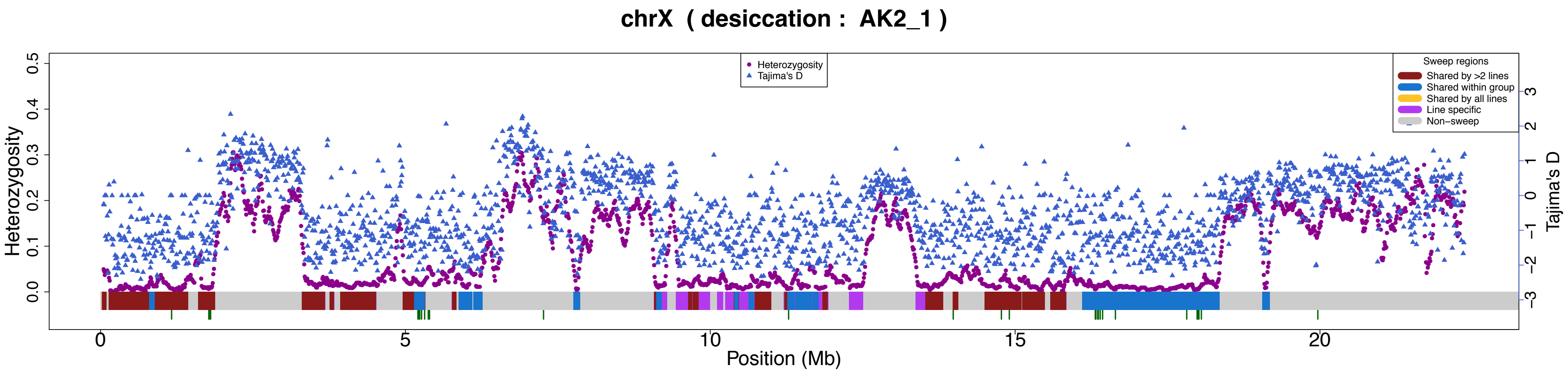
chrX (desiccation : AK2_1)
Heterozygosity
Tajima's D
Sweep regions
Shared by >2 lines
Shared within group
Shared by all lines
Line specific
Non–sweep
Heterozygosity
Tajima's D
Position (Mb)
0
5
10
15
20
0.0
0.1
0.2
0.3
0.4
0.5
−3
−2
−1
0
1
2
3

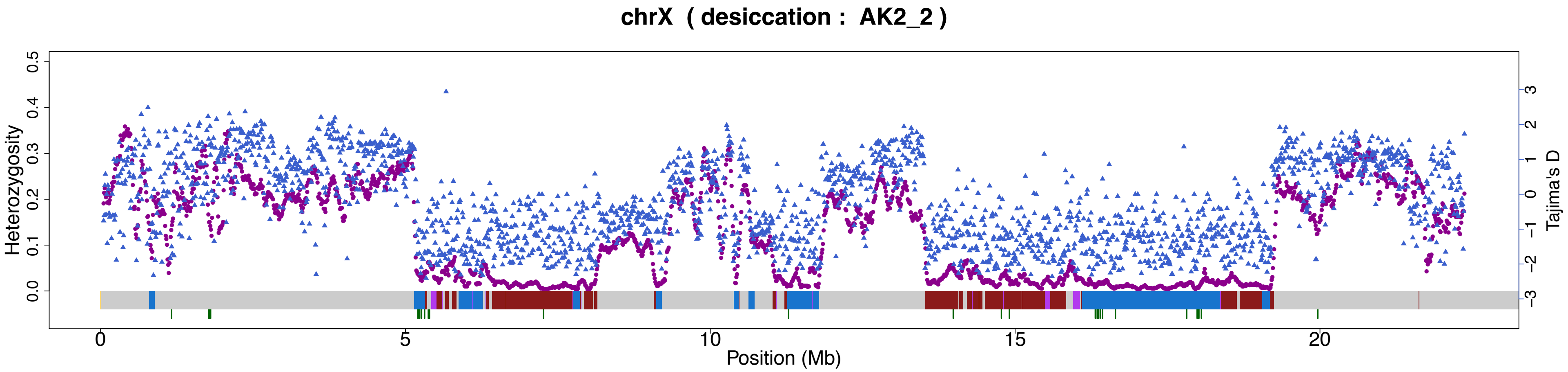
chrX (desiccation : AK2_2)
Heterozygosity
Tajima's D
Position (Mb)
0
5
10
15
20
0.0
0.1
0.2
0.3
0.4
0.5
−3
−2
−1
0
1
2
3

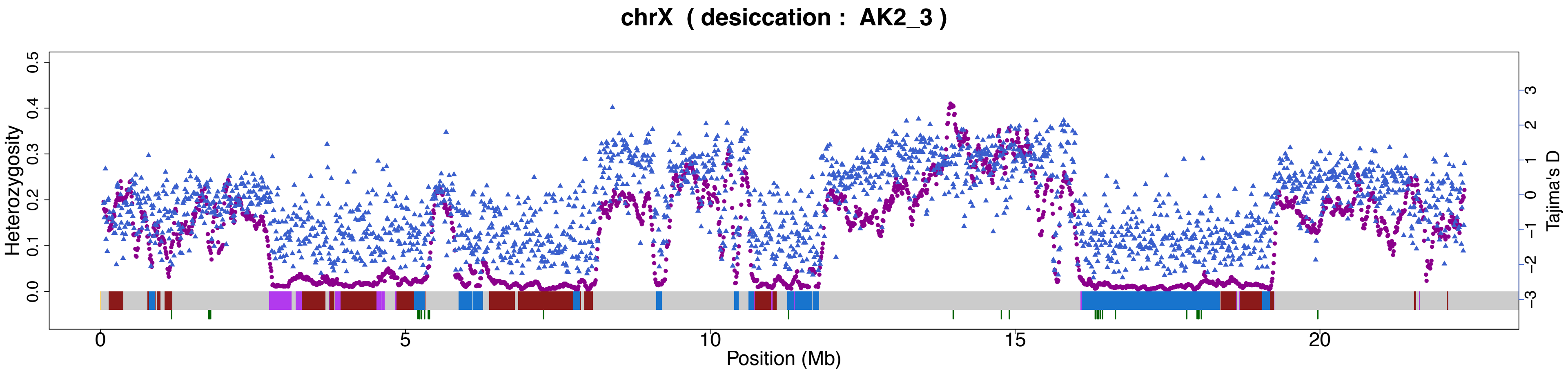
chrX (desiccation : AK2_3)
Heterozygosity
Tajima's D
Position (Mb)
0
5
10
15
20
0.0
0.1
0.2
0.3
0.4
0.5
−3
−2
−1
0
1
2
3

chrX (control : AK2_4)

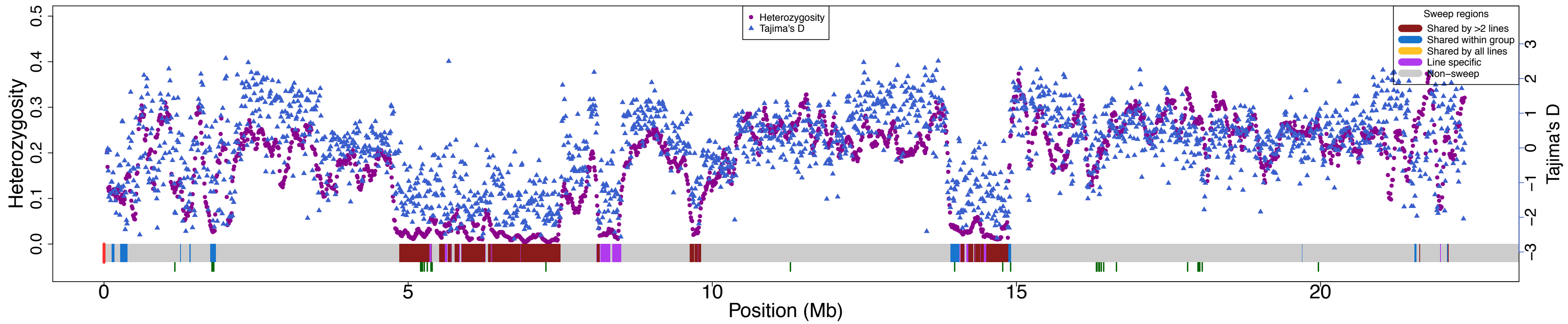

chrX (control : AK2_7)

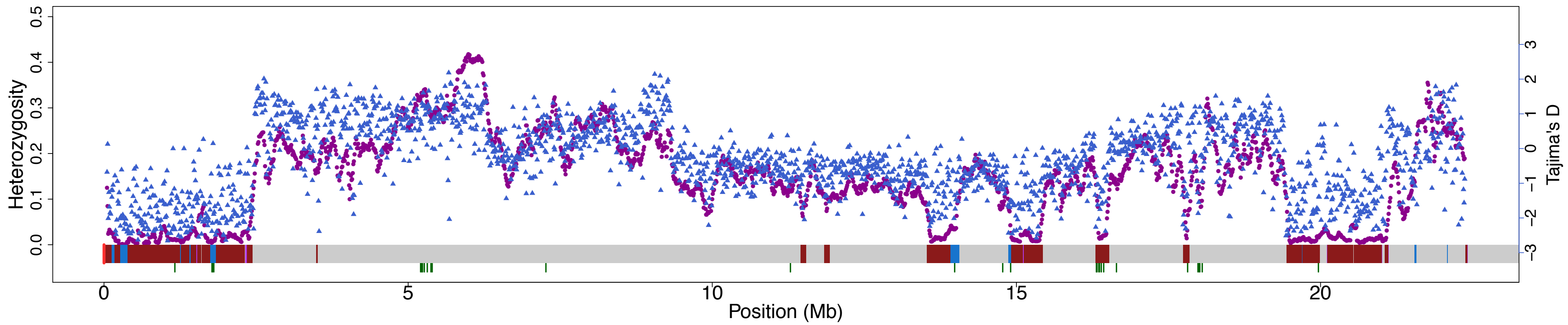

chrX (control : AK2_8)

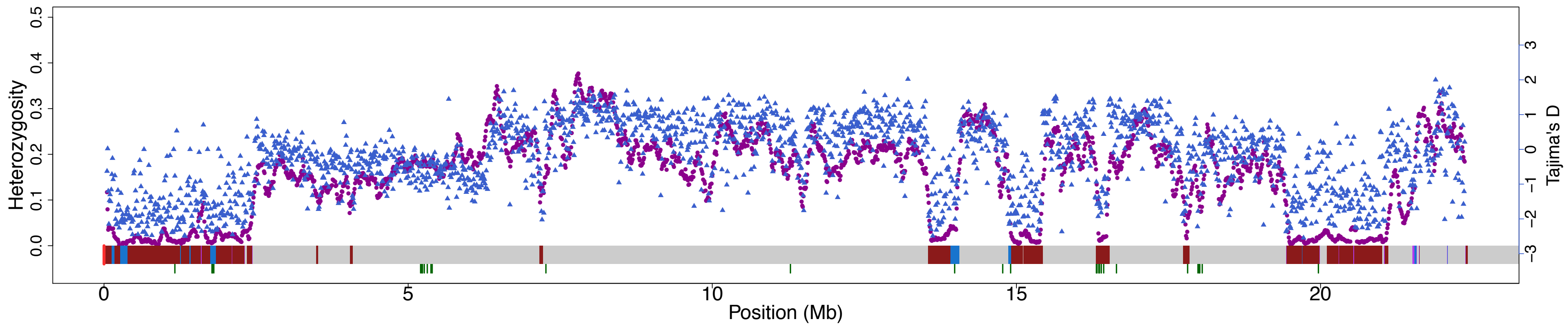

Supplement: Additional file 5: — Figures of heterozygosity and Tajima’s D values plotted against the putative selective sweep signatures (horizontal color blocks) along chromosomal 2 L, 2R, 3R, and X. (PDF 2138 kb) [file 12864_2016_2556_MOESM5_ESM.pdf]
